# Mitochondrial genome sequencing and phylogeny of *Haemagogus albomaculatus*, *Haemagogus leucocelaenus*, *Haemagogus spegazzinii*, and *Haemagogus tropicalis* (Diptera: Culicidae)

**DOI:** 10.1038/s41598-020-73790-x

**Published:** 2020-10-12

**Authors:** Fábio Silva da Silva, Ana Cecília Ribeiro Cruz, Daniele Barbosa de Almeida Medeiros, Sandro Patroca da Silva, Márcio Roberto Teixeira Nunes, Lívia Carício Martins, Jannifer Oliveira Chiang, Poliana da Silva Lemos, Gabriel Muricy Cunha, Renato Freitas de Araujo, Hamilton Antônio de Oliveira Monteiro, Joaquim Pinto Nunes Neto

**Affiliations:** 1 Graduate Program in Parasitary Biology in the Amazon, Center of Biological and Health Sciences, State University of Pará, Belém, 66095-662 Brazil; 2 Arbovirology and Hemorrhagic Fevers Section, Evandro Chagas Institute – IEC/MS/SVS, Ananindeua, 67030-000 Brazil; 3 Center for Technological Innovation, Evandro Chagas Institute – IEC/MS/SVS, Ananindeua, 67030-000 Brazil; 4 Directorate of Epidemiological Surveillance, Bahia State Department of Health, Salvador, 40279-700 Brazil

**Keywords:** Mitochondrial genome, Entomology

## Abstract

The genus *Haemagogus* (Diptera: Culicidae) comprises species of great epidemiological relevance, involved in transmission cycles of the *Yellow fever virus* and other arboviruses in South America. So far, only *Haemagogus janthinomys* has complete mitochondrial sequences available. Given the unavailability of information related to aspects of the evolutionary biology and molecular taxonomy of this genus, we report here, the first sequencing of the mitogenomes of *Haemagogus albomaculatus*, *Haemagogus leucocelaenus*, *Haemagogus spegazzinii*, and *Haemagogus tropicalis*. The mitogenomes showed an average length of 15,038 bp, average AT content of 79.3%, positive AT-skews, negative GC-skews, and comprised 37 functional subunits (13 PCGs, 22 tRNA, and 02 rRNA). The PCGs showed ATN as start codon, TAA as stop codon, and signs of purifying selection. The tRNAs had the typical leaf clover structure, except *tRNA*^*Ser1*^. Phylogenetic analyzes of Bayesian inference and Maximum Likelihood, based on concatenated sequences from all 13 PCGs, produced identical topologies and strongly supported the monophyletic relationship between the *Haemagogus* and *Conopostegus* subgenera, and corroborated with the known taxonomic classification of the evaluated taxa, based on external morphological aspects. The information produced on the mitogenomes of the *Haemagogus* species evaluated here may be useful in carrying out future taxonomic and evolutionary studies of the genus.

## Introduction

The genus *Haemagogus* Williston, 1896 (Diptera: Culicidae), belongs to the Aedini tribe and comprises 28 species of mosquitoes with occurrence records restricted to the Neotropical region, and widely distributed throughout Central and South America^1^. Currently, it includes two subgenera: *Conopostegus* Dyar, 1925, with four species, and *Haemagogus* Williston, 1896, with 24 species^1–3^. The species belonging to the latter subgeneric category are subdivided into three sections: Albomaculatus (14 species), Splendens (09 species), and Tropicalis (01 species)^1^. In Brazil, the occurrence of nine species of *Haemagogus* has been reported, with representatives for both subgenera and Sects. ^4^. They are wild mosquitoes, with daytime habits, not commonly found outside the forest environment^2^. However, some species had shown a great adaptive capacity, due to environmental degradation conditions, invading houses, especially those located near areas of altered forest^5,6^.

*Haemagogus* has great medical-epidemiological relevance, due to the involvement of some species in the transmission and maintenance of important arboviruses. In South America, *Haemagogus janthinomys* Dyar, 1925 is the main vector of the *Yellow Fever virus* (YFV) in the wild cycle and has a widespread distribution in the Brazilian territory^4,7,8^. Moreover, it is also the main vector for the *Mayaro virus* (MAYV), an alphavirus endemic in the Amazon rainforest^9,10^. *Haemagogus leucocelaenus* Dyar & Shannon, 1924 is also an important vector in the transmission of YFV, mainly in the South and Southeast of Brazil^11,12^. Other species are also related to secondary YFV transmission in the country, such as *Haemagogus albomaculatus* Theobald, 1903^13,14^, *Haemagogus spegazzinii* Brèthes, 1912^15^, and *Haemagogus tropicalis* Cerqueira & Antunes, 1938^16^. This latter species is restricted to the Brazilian Amazon region^1,17,18^.

The use of genomic sequencing tools in studies of evolutionary and molecular taxonomy, in particular, of arthropods of epidemiological importance, has significantly contributed to the provision of critical information useful for vector research and control strategies, allowing not only an improved visualization of the evolutionary and phylogenetic patterns of these organisms, but also contributing directly to the subsidy regarding co-evolutionary properties between these and the infectious agents that they can harbor and potentially transmit^19–25^.

The mitochondrial genome (mitogenome) in most metazoan organisms is a small double-stranded DNA molecule, ranging from 15 to 20 kb in length and contain 37 functional subunits, of which 13 are protein-coding genes (PCGs), 22 are transfer RNA (tRNA), and two are ribosomal RNA (*rRNA*^*12*^^*S*^ and *rRNA*^*16S*^). Besides, it also has a region rich in Adenine and Thymine (A + T), associated with the control of the molecule's replicative processes^26^. Due to factors such as the absence of recombinant processes and the high rate of evolution and accumulation of mutations concerning the nuclear genome, the mitogenome stands out as one of the main markers used for conducting evolutionary and taxonomic studies of vertebrates and invertebrates^27–30^. The use of mitochondrial sequences in evolutionary studies, in particular of insects, has produced significant results in Diptera^31–33^, Hemiptera^34^, Hymenoptera^35^, and Lepidoptera^36^.

Despite the great medical-epidemiological importance of *Haemagogus*, there is still a great unavailability of data and information on the evolutionary biology and molecular taxonomy aspects of this genus. The only species of this genus, so far, that has a complete sequencing data for the mitogenome is *Hg. janthinomys*^37^. In addition, only partial sequences of the *rRNA*^*16S*^ and *COI* genes of *Hg. janthinomys*, *Hg. leucocelaenus*, *Hg. spegazzinii*, *Haemagogus lucifer* Howard, Dyar & Knab, 1913, and *Haemagogus equinus* Theobald, 1903 are available on the Genbank database.

Considering the great potential of mitochondrial genes in conducting taxonomic and phylogenetic studies, we performed, for the first time, the characterization of mitogenomes, using the High Troughtput Sequencing (HTS) of the species *Hg. albomaculatus*, *Hg. leucocelaenus*, *Hg. spegazzinii*, and *Hg. tropicalis*. The four mitogenomes obtained were analyzed, compared to those of other species of the Culicidae family available on GenBank database, and the phylogeny was reconstructed based on the concatenation of all 13 PCGs by Bayesian inference and Maximum Likelihood methods.

## Results and discussion

### Organization and composition of the obtained mitogenomes

**Figure 1:**
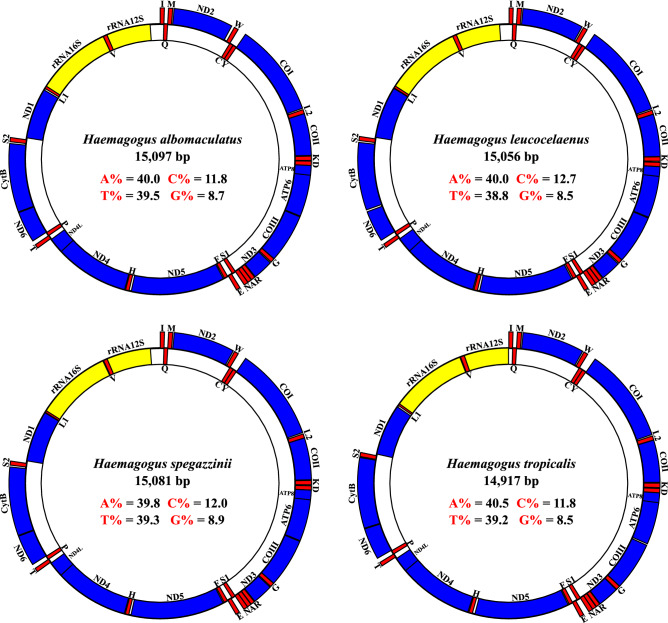
Structural representations of the *Hg. albomaculatus*, *Hg. leucocelaenus*, *Hg. spegazzinii*, and *Hg. tropicalis* mitogenomes. The internal values indicate the content of the nucleotide bases. The blue, red, and yellow blocks indicate PCGs, tRNAs, and rRNAs, respectively. Each tRNA is identified by a unique letter abbreviation. The genes arranged in the outer circle are located in the J strand (Foward), and the genes arranged in the inner circle are located in the N strand (Reverse). The structural representations of the genomes in this figure were generated using *GenomeVx* (https://wolfe.ucd.ie/GenomeVx/) and edited using *Inkscape 0.92.4* (https://inkscape.org/).

The *Haemagogus* mitogenomes obtained (Supplementary Tables 3–6) were structured into compact circular double-stranded DNA molecules, with lengths ranging from 14,917 bp (*Hg. tropicalis*) to 15,097 bp (*Hg. albomaculatus*), and with total AT contents ranging from 78.8% (*Hg. leucocelaenus*) to 79.7% (*Hg. tropicalis*). They also comprised 37 functional and conserved subunits—13 PCGs, 22 tRNAs, and 02 rRNAs—and demonstrated similar patterns of gene positioning along the J (Foward) and N (Reverse) strands (Fig. 1), similarly to that observed in studies of other species of Culicidae^32,37–45^, and other groups of insects^31,46–48^ with reported mitogenomes.

Asymmetry analyzes of the total genome of each species evaluated resulted in positive AT-skews (average of 0.0108) and negative GC-skews (average of − 0.1659) (Supplementary Table 7A), showing the content of Adenine and Cytosine in greater proportions in the majority chain compared to the content of Thymine and Guanine, respectively. Those findings corroborate the pattern of nucleotide composition observed in other groups of insects^31,46,49^, and were similar to previous studies for *Anopheles*^32,44,50,51^, *Culex*, and *Lutzia*^39,45^.

**Figure 2:**
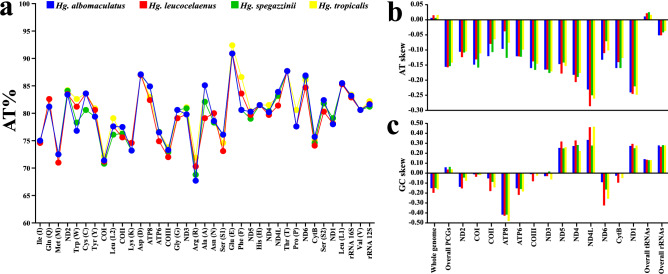
Information on lengths, AT contents, and AT/GC-skews of the investigated mitogenomes. AT contents (%) (**a**). AT-skews (**b**). GC-skews (**c**). All graphs in this figure were generated using *GraphPad Prism 8.0.1* (https://www.graphpad.com/scientific-software/prism/) and edited using *Inkscape 0.92.4* (https://inkscape.org/).

In PCGs, the total length ranged from 10,917 bp (*Hg. albomaculatus*) to 10,950 bp (*Hg. spegazzinii*), and the AT content varied from 77.4% (*Hg. leucocelaenus*) to 78.4% (*Hg. tropicalis*) (Supplementary Table 7A). Similar to that reported for other species of Culicidae^39,40,52^, in this study, the AT content increased considerably at the position of the third codon in these regions, ranging from 93.5% (*Hg. leucocelaenus*) to 96.1% (*Hg. albomaculatus*) (Supplementary Table 7B). This increase in the AT content may be related to events where there is a high availability of cellular ATP, parallel to a decrease in other NTPs, thus promoting an increase in the efficiency of the transcription due to the maximization, mainly of Adenine, in the position of the third codon^53^. The asymmetry analysis of these concatenated regions resulted in negative AT-skews (average of − 0.1521) and positive GC-skews (average of 0.0491) (Supplementary Table 7A). The higher proportions of Thymine and Guanine in relation to Adenine and Cytosine in these regions contrasted with the values of asymmetry obtained in the evaluation of complete sequences, similar to that reported in other studies of Culicidae^40,44,54^. Individually assessed, PCGs presented average AT contents ranging from 71.4% (*COI*) to 86.1% (*ND6*) (Fig. 2a). Also, all PCGs had negative AT-skews (Fig. 2b) and, in most cases, negative GC-skews, except *COIII* and *ND3* (only in *Hg. spegazzinii*), *ND5*, *ND4*, *ND4L*, and *ND1* (Fig. 2c), as observed in other studies^44,52^.

The mitogenomes obtained here presented sets of 22 tRNAs, with approximate values in total lengths (average of 1488 bp), AT contents (average of 80.2%), and positive AT/GC-skews (Supplementary Table 7A), similar to that reported for other species of Culicidae^40,44,54,55^. Individually, the average AT content in these regions ranged from 69.7% (*tRNA*^*Arg*^) to 91.3% (*tRNA*^*Glu*^) (Fig. 2a), and the average lengths ranged from 64 bp (*tRNA*^*Arg*^) to 72 bp (*tRNA*^*Val*^) (Supplementary Tables 3–6). The typical cloverleaf-like structure, including four arms [Amino acid acceptor (AA), dihydrouridine (DHU), T_Ψ_C, and Anticodons (AC)] and loops [AA, DHU, T_Ψ_C, and Variable (V)] was observed in 21 of the 22 tRNAs described for the sequenced species (Supplementary Figs. 1–4). The *tRNA*^*Ser1*^ was the only one that did not present a secondary structure similar to the others, showing the absence of DHU arm, replaced by a DHU loop, similar to that reported in previous studies of other mosquito species^32,39,44,54^, and other groups of insects^31,48,56^. Besides, between 18 and 22 mismatched base pairs were detected in the tRNAs of the mitogenomes sequenced: in *Hg. albomaculatus*, there are 17 GU pairs and 01 UU; in *Hg. leucocelaenus*, there are 20 GU pairs and 02 UU; in *Hg. spegazzinii*, there are 19 GU pairs and 01 UU; and in *Hg. tropicalis*, there are 16 GU pairs and 02 UU.

The subunits *rRNA*^*16*^^*S*^ and *rRNA*^*12S*^, concatenated, had lengths (average of 2,153 bp) and AT contents (average of 82.6%) (Supplementary Table 7A), similar in the four mitogenomes obtained, with negative AT-skews (Fig. 2b) and positive GC-skews (Fig. 2c). Individually, they presented, respectively, average AT contents of 83.2% and 81.7%, and average lengths of 1,360 bp and 792 bp (Supplementary Tables 3–6).

The mitochondrial sequences obtained presented between 24 and 25 small intergenic regions, non-coding, and with sizes varying from 1 to 47 nucleotides, totaling in *Hg. albomaculatus* 394 non-coding nucleotides; in *Hg. leucocelaenus*, 351; in *Hg. spegazzinii*, 367; and in *Hg. tropicalis*, 387 (Supplementary Tables 3–6). In the first three species, there is an intergenic spacer with an average length of 174 nucleotides, located between *rRNA*^*12*^^*S*^ and *tRNA*^*Ile*^. In this location, probably, the control region (A + T) of these species would be located^57^, as predicted in other Culicidae studies^32,41,45,54^, and other dipterans^31,46,47^. In *Hg. tropicalis*, there is an intergenic spacer of only 5 bp between the two subunits mentioned.

In this study, no sequencing data were obtained for the control region (A + T) of the analyzed species. In metazoans, this region is associated with the initial processes of mitogenome transcription and replication, presenting, in particular in invertebrates, variable lengths due to factors such as high rates of nucleotide substitution, insertions/deletions, and a varied number of repetitions (homopolymers)^57–59^. Likewise, Lemos et al*.*^37^, sequencing the mitogenome of *Hg. janthinomys* obtained a nucleotide sequence (length of 14.937 bp) with the absence of this region. It is essential to adopt methods that aim to include information about the control region of the species analyzed in future studies. Techniques such as conventional *PCR* and sequencing by the Sanger method are relevant alternatives that can be used in an attempt to obtain these data, as they allow selective amplification of specific mitogenome regions, with satisfactory results in similar studies^60–62^.

### Characteristics of protein-coding genes (PCGs)

In the mitogenomes obtained in this study, nine PCGs (*ATP6*, *ATP8*, *COI*, *COII*, *COIII*, *CytB*, *ND2*, *ND3*, and *ND6*) showed a sense of transcription on J strand (Foward), and four (*ND1*, *ND4*, *ND4L*, and *ND5*) on N strand (Reverse). Except for *ATP6* (which used TTG as the start codon), the other PCGs, in the four mitogenomes, used the ATN standard as the start codon (Supplementary Tables 3–6), similar to that reported in other studies with Culicidae^32,39,45,54^.

In the genes *ND1*, *ND2*, *ND3*, and *ND6*, all species are using ATA as the start codon. In *Hg. albomaculatus*, *Hg. leucocelaenus*, and *Hg. spegazzinii*, the sets of PCGs [*ATP8*, *COI*, *CytB* and *ND4*], [*COII* and *COIII*], and [*ND4L* and *ND5*] used, respectively, the start codons ATT, ATG, and ATC. In *Hg. tropicalis*, the sets of PCGs [*ATP8*, *COI,* and *COII*], [*COIII, CytB, ND4*, and *ND4L*], and [*ND5*] they also used, respectively, the start codons ATT, ATG, and ATC. The complete stop codon TAA was common to all PCGs. It is estimated that the expression of complete TAA stop codons cans be related to post-transcriptional polyadenylation events^63^, and although it was not detected in this study, the recording of incomplete stop codons (T/TA) is common in insects^32,45,55,58,64^.

**Figure 3:**
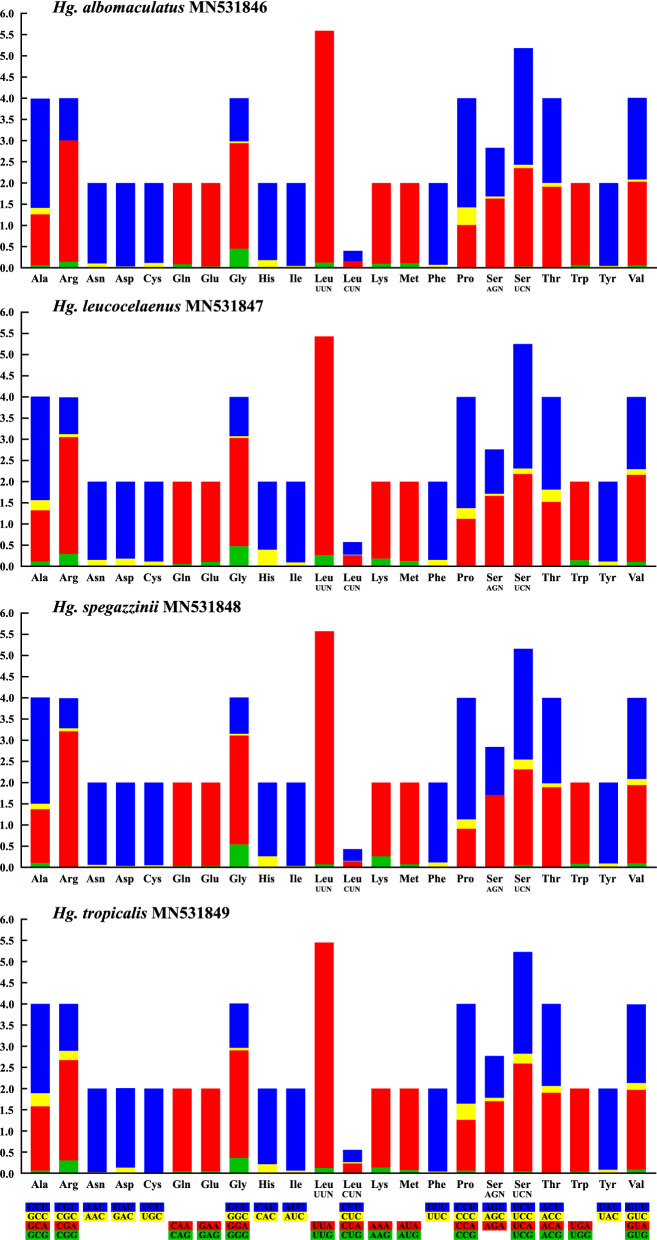
Relative synonymous codon usage (RSCU) of the sequenced mitogenomes. RSCU values are represented on the y-axis, and families of synonymous codons and their respective amino acids are indicated on the x-axis. All graphs in this figure were generated using *GraphPad Prism 8.0.1* (https://www.graphpad.com/scientific-software/prism/) and edited using *Inkscape 0.92.4* (https://inkscape.org/).

Excluding stop codons, there are 3,639 codons in use in *Hg. albomaculatus*, 3,647 in *Hg. leucocelaenus*, 3,650 in *Hg. spegazzinii*, and 3,646 in *Hg. tropicalis* mitogenomes. The analysis of the RSCU (Supplementary Table 8) showed that almost all codons are present in the sequenced mitogenomes, with the exception of AGG, which synthesizes the Serine amino acid. In addition, the codons with Adenine and/or Thymine (Uracil) in the third position were much more used in comparison with other synonymous codons, with Cytosine and/or Guanine in the third position (Fig. 3). This can be seen, for example, in the use of the amino acid Leucine (L), which presented the codons with the highest and lowest frequency of use in the four newly-sequenced mitogenomes: while UUA had an average RSCU value of 5.37, CUG, which synthesizes the same amino acid, had an average RSCU of 0.01. Thus, among the four investigated mitogenomes, the most frequently used codons were, in decreasing order, UUA (L) (average RSCU value 5.37), CGA (A) (2.80), UCU (S) (2.68), CCU (P) (2.61), and GGA (G) (2.54), while CUC (L) (RSCU value 0.01), ACG (T) (0.01), and CUG (L) (0.01) were rarely used. It was observed that all codons with RSCU ≥ 1 were of the NNU and/or NNA type, corroborating with other studies, in which this tendency to use codons seems to follow a frequent pattern, as observed in Culicidae^39,40,44,45,50,54^, and other groups of insects^33,36,56^. Among the four sequenced mitogenomes, a total of 20 different amino acids are coded, with Leucine being the most frequent amino acid (15.9%), and Cysteine the least frequent (1.01%).

### Evolutionary selection analysis

**Figure 4:**
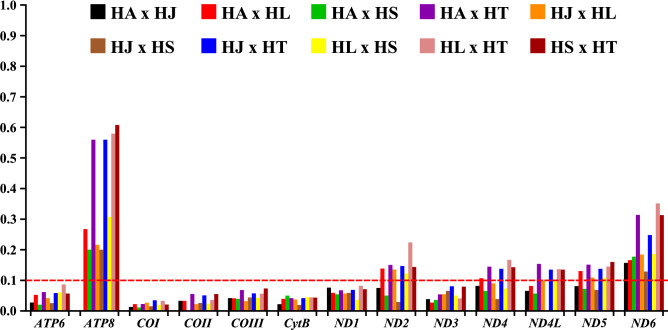
Proportions between rates non-synonymous (*d*_*N*_) and synonymous (*d*_*S*_) nucleotide substitutions (*d*_*N*_*/d*_*S*_) in 05 *Haemagogus* mitogenomes. The ratios were calculated in paired analyzes between the 13 PCGs of the sequenced mitogenomes, together with the data already available for *Hg. janthinomys*. The *d*_*N*_*/d*_*S*_ ratios are plotted on the y-axis, and the PCGs are plotted on the x-axis. Abbreviations: HA (*Hg. albomaculatus*), HJ (*Hg. janthinomys*), HL (*Hg. leucocelaenus*), HS (*Hg. spegazzinii*), and HT (*Hg. tropicalis*). The graph in this figure was generated using *GraphPad Prism 8.0.1* (https://www.graphpad.com/scientific-software/prism/) and edited using *Inkscape 0.92.4* (https://inkscape.org/).

In order to evaluate the pressure of evolutionary selection acting on the PCGs of the sequenced mitogenomes in this study, together with *Hg. Janthinomys* (GenBank ID: NC28025), pairwise analyzes were performed to estimate the proportions between the existing non-synonymous (*d*_*N*_) and synonymous (*d*_*S*_) substitutions. The results obtained indicate that the 13 PCGs in the analyzed mitogenomes are globally evolving under negative selection pressure (purification) (*d*_*N*_*/d*_*S*_ < 1), with ratios ranging from 0.0115 ± 0.0347 in *COI* to 0.0 ± 0.6079 in *ATP8* (Fig. 4), displaying the following order: *COI* < *COII* < *CytB* < *ATP6* < *COIII* < *ND3* < *ND1* < *ND4L* < *ND4* < *ND5* < *ND2* < *ND6* < *ATP8*. The purifying selection was particularly strong (*d*_*N*_*/d*_*S*_ < 0.1) in the first seven coding regions of the order presented, with greater emphasis on the genes of complex III (*CytB*) and IV (*COI*, *COII*, and *COIII*) on mitogenomes. At the same time, the complex I genes (*NADH*), together with *ATP8*, showed higher *d*_*N*_*/d*_*S*_ proportions compared to the other genes, which indicates the presence of less conservative evolutionary restrictions in these regions, wich showed a relaxed purifying selection. The rates of synonymous substitutions were significantly higher compared to non-synonymous substitutions in all PCGs (mainly in the *COI*) of the *Haemagogus* mitogenomes evaluated here. The results obtained corroborate the pattern observed in previous studies, which also demonstrated the existence of a heterogeneity between the evolution rates in the different complexes that encode proteins of the mitochondrial genome, as well as the predominance of a strong active purifying selection in these regions^32,39,55,65^.

### Nucleotide diversity analysis

**Figure 5:**
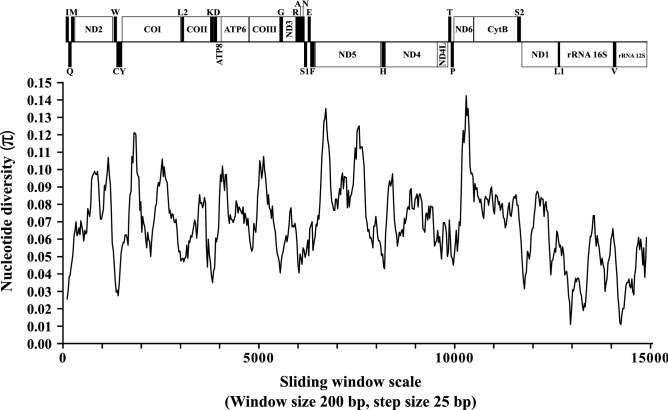
Nucleotide diversity among the mitogenomes of the four species evaluated, with *Hg. janthinomys* (GenBank ID: NC28025). The π values were calculated from a sliding window analysis of 200 bp in 25 bp steps, and are represented on the y-axis. The length values of the aligned sequence are represented on the x-axis. The limits of each gene are indicated in the representation above: vertical black bars indicate tRNAs and white rectangles indicate PCGs and rRNAs. The graph of this figure was generated using *DnaSP v.6.12.03* (https://www.ub.edu/dnasp/) and edited using *Inkscape 0.92.4* (https://inkscape.org/).

We performed here a sliding window analysis based on the complete alignment of the mitogenomes sequenced in this study, together with the data already available in *Hg. janthinomys* (GenBank ID: NC28025), in order to verify which regions of the mitogenome have greater nucleotide divergence, allowing the identification of potential markers that can be used in future evolutionary investigations of the *Haemagogus* genus. The results demonstrated a diversity of nucleotides (π) ranging from 0.0110 to 0.1425 between the sequences analyzed (Fig. 5). Among the PCGs (all showed π ≥ 0.06), the highest values of diversity (π ≥ 0.11) were for *ND6* (π  = 0.0450 ± 0.1425), *ND5* (π  = 0.0430 ± 0.1350), and *COI* (π  = 0.0275 ± 0.1210). Just below (0.11 > π  ≥ 0.10), were *COIII* (π  = 0.0405 ± 0.1075), *ND2* (π  = 0.0305 ± 0.1070), and *ATP6* (π  = 0.0550 ± 0.1020). Among tRNAs, the highest diversity value (0.09 > π  ≥ 0.08) was for *tRNA*^*Ser2*^ (π  = 0.0315 ± 0.0855), and for rRNAs, the highest diversity value achieved (0.08 > π  ≥ 0.06) went to *rRNA*^*16S*^ (π  = 0.0110 ± 0.0735), followed by *rRNA*^*12S*^ (π  = 0.0110 ± 0.0610).

Notably, PCGs showed the highest nucleotide diversity values, compared to those obtained for tRNAs and rRNAs. Among these, the 5′ proximal ends of the *ND5* and *COI* genes showed two of the most divergent points, similar to those observed in other other descriptive studies focusing on the mitogenome of other mosquitoes species^39,40,52^. In particular, the *COI* gene is widely used in phylogenetic studies, being used as a universal barcode for species identification^66^. Two of the main advantages of using this gene are: the high robustness and availability of universal primers that allow to recover the 5′ end of the representatives of most animal phyla^67^, and, being present in hundreds of copies per cell, like other PCGs, this gene has a high rate of base replacement, especially in the third codon region. However, it is observed that changes in its amino acid sequence seem to occur more slowly than in any other mitochondrial gene, a factor that directly contributes to the resolution of deeper taxonomic affinities^66,68^. In mosquitoes, species identification is difficult in some cases due to the great similarity of the external anatomical characteristics. In this situation, sometimes only the observation of structures of the male genitalia allows a reliable identification^69^. In this sense, the alternative use of the *COI* in the identification of species of great epidemiological importance has shown satisfactory results^70–75^.

The results obtained here suggest that PCGs are the most suitable regions to be used as molecular markers to elucidate the phylogenetic relationships between sequenced species of *Haemagogus*. More specifically, the combined results of the sliding window analysis and the *d*_*N*_*/d*_*S*_ ratios suggest that the *COI*, *ND5*, *ND6*, and *ATP8* genes represent potential evolutionary markers in future studies on *Haemagogus* genus, based on the highest values of nucleotide diversity (in *COI*, *ND5*, and *ND6*), in the strong purifying selection that operates in these regions, especially in *COI*, and in the record of the highest accumulation rates of non-synonymous substitutions in *ATP8* and *ND6*, compared to other PCGs. However, these results need to be evaluated in future studies with the inclusion of a greater number of mitochondrial sequences from other species of the genus investigate.

### Phylogenetic analysis

The nucleotide sequences alignment containing all 13 PCGs of 27 taxa (04 of them with mitogenomes sequenced in this study, and another 23 mitogenomes with data available in the GenBank database), showed an average nucleotide distance of 0.16%, with percentages ranging from 0.01 to 0.28%. The *Hg. janthinomys* (GenBank ID: NC028025) was the taxon with the lowest average nucleotide distance (0.07%), and *Dixella aestivalis* (GenBank ID: NC029354) was the taxon with the highest average nucleotide distance (0.26%), when compared to the newly-sequenced species (Supplementary Table 9).

**Figure 6:**
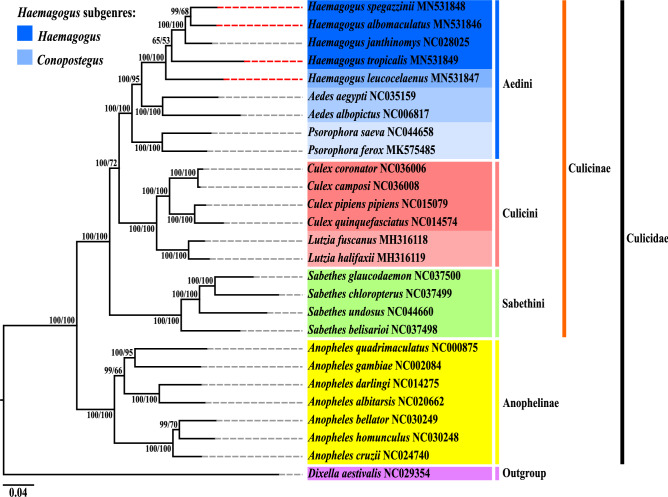
Phylogenetic reconstruction by the Bayesian Inference and Maximum Likelihood methods, based on the concatenation of the 13 PCGs of the *Haemagogus* species sequenced in this study and 23 other taxa with data available on GenBank database. The subsequent Bayesian probabilities (BP), and the values of support for bootstrapping (BPP) for 1,000 replicates, respectively, are shown on the left, in each node. The complementary lines dotted in red indicate the newly-sequenced species. The GenBank database accession numbers and references of mitogenomes sequences of the taxa used are listed in Supplementary Table 2. The topologies were visualized using *FigTree v.1.4.4* (https://tree.bio.ed.ac.uk/software/figtree/) and edited using *Inkscape 0.92.4* (https://inkscape.org/).

The best nucleotide replacement model defined under the Akaike information criterion (AIC) for the reconstruction of phylogenies by Bayesian inference and Maximum Likelihood methods was the *General Time Reversible with Gamma Distribution* (*GTR* + *G*). The phylogenetic analyzes by the two methods produced topologies of identical trees (Fig. 6; Supplementary Figs. 5, 6), with an initial structure of 26 taxa in a large monophyletic group, corresponding to the Culicidae family, anchored externally by the taxon *Dixella aestivalis* (Dixidae). The definition of the external group in the two phylogenies occurred in an automated way (*midpoint*)^76^.

The phylogeny obtained showed that Culicidae contained two well-related clades (BP/BPP = 100%/100%), corresponding to the subfamilies Anophelinae and Culicinae. Anophelinae, containing seven taxa, demonstrated a monophyletic conformation previously predicted in morphological and molecular studies, focusing on the mitogenomes of species of the *Anopheles* genus^51,77–79^. Likewise, it was observed in Culicinae a monophyletic arrangement formed by 19 taxa distributed in three subclades, which corroborated with studies of morphological and molecular taxonomy, also based on the sequencing of the mitogenome of species belonging to the tribes Sabethini^42,43^, Culicini^39,40,45^, and, in particular, Aedini^2,37,80^, which in the phylogeny reconstructed, included three closely related groupings, corresponding to the *Haemagogus*, *Aedes*, and *Psorophora* genera.

Currently, the *Haemagogus* genus, focus of this study, comprises 28 species, distributed in two subgenera: *Conopostegus*, with 04 species, and *Haemagogus*, with 24 species. The current internal taxonomic classification of this genus, based on morphological aspects, is mainly due to the review studies carried out by Zavortink^3^, who proposed the relocation of *Hg. leucocelaenus* and three other species representatives of the subgenus *Conopostegus*, previously classified in *Aedes*, to the *Haemagogus* genus, and by Arnell^1^, who supported the relocation proposed by Zavortink^3^ and proposed the merging of all other 24 species, previously classified into three subgenera (*Haemagogus*, *Stegoconops* Lutz, 1905, and *Longipalpifer* Levi-Castillo, 1951) in a single subgenus (*Haemagogus*). In addition, Arnell^1^ also proposed to subdivide the species of the newly merged subgenus *Haemagogus* into three sections: Albomaculatus, with 14 species, would contain all those previously belonging to the old subgenera *Stegoconops* and *Longipalpifer*; Splendens, with 09 species, would contain those previously belonging to the old subgenus *Haemagogus*; and Tropicalis, which would be monotypic, comprising only the species *Hg. tropicalis*, which has morphological characters from the first section, in the adult phase, and the second, in the larval development phase.

The phylogeny of *Haemagogus* spp. resulted in a monophyletic subclade, in accordance with the taxonomic classification known for the genus^1–3,80^. The generated topology allowed the taxa classification into two subgenera, equally to the current taxonomic classification based on morphology: *Hg. leucocelaenus*, the first species among the taxa evaluated to compose the evolutionary lineage of the grouping, belongs to the subgenus *Conopostegus*, while the other taxa in the above branches belong to the subgenus *Haemagogus*. For this last subgeneric category, it was also possible to classify taxa into two species sections: *Hg. albomaculatus*, *Hg. spegazzinii*, and *Hg. janthinomys* belong to the Albomaculatus section, and just below *Hg. tropicalis* belongs to the monotypic section Tropicalis^1^. The low nucleotide distance observed among *Haemagogus* sequences (which ranged from 0.05 between *Hg. albomaculatus* and *Hg. spegazzinii* to 0.11 between *Hg. leucocelaenus* and *Hg. Tropicalis*—Supplementary Table 9) supports the monophilia observed in the structuring of the genus subclade.

The results of the reconstructed phylogeny strongly corroborated the review study carried out by Zavortink^3^, were this subgenus maintained many primitive morphological characters and would probably be closer to the ancestral lineage of the genus. In addition, more recent studies have made observations on *Hg. leucocelaenus*, that demonstrated, in addition to the morphological similarity, ecological behaviors similar to the species of *Aedes*^6,11,12^. Therefore, based on the mitochondrial genes used and the number of available taxa, it was possible to confirm the current evolutionary position of this species and its subgenus.

*Haemagogus tropicalis* presented itself as the first species in the evolutionary lineage of the group of taxa belonging to the subgenus *Haemagogus*. Despite the taxonomic agreement, the probability of anchoring this taxon in relation to the upper grouping was low in the two phylogenetic analysis methods applied (BP/BPP = 65%/53%), considering the number of available taxa and included in the analysis. It is important to note that, in this study, mitogenome sequencing data were obtained only from representatives of the sections Albomaculatus and Tropicalis of the subgenus *Haemagogus*, lacking genomic data from representatives of the Splendens section. The future inclusion of more taxa may likely influence the phylogenetic relations of the mitochondrial genes used, however, it will be of great relevance for the evolutionary biology studies of the genus investigated.

To address the effectiveness of the genes *ATP8*, *COI*, *ND5*, and *ND6* in the reconstruction of the phylogenetic relationships of the newly-sequenced species, an additional phylogenetic analysis was performed adopting the same parameters established for the phylogeny based on the use of 13 PCGs (Supplementary Figs. 7, 8). The topologies generated for the *Haemagogus* species grouping were similar to those obtained in the 13 PCGs.

## Methods

### Sampling and extraction of total DNA

The *Haemagogus* samples used in this study came from field expeditions carried out in the state of Bahia (Northeast Brazil), in the regions of Jaborandi (S 13° 58′ 92′' W 44° 53′ 36″) in 2013, and Coribe (S 13° 82′ 94″ W 44° 44′ 92″), and in the state of Pará (Northern Brazil), in the regions of Canãa dos Carajás (S 06° 49′ 75″ W 49° 87′ 83″) in 2016 (MMA/ICMbio/Carajás National Forest/Direct authorization nº021/2018), and Ilha do Combu (S 01° 51′ 97″ W 48° 49′ 28″) in 2018 (MMA/ICMBio/SISBIO authorization nº60595-1) (Supplementary Table 1). The specimens were identified taxonomically using gender-specific dichotomous keys^2,4,81^, organized in batches containing mosquitoes of the same species, and stored at − 70ºC for conservation. Subsequently, the mosquito batches were macerated with 3 mm stainless stell beads using TissueLyser II (Qiagen), and the genomic DNA of each batch of species was extracted*,* using the DNeasy Blood & Tissue kit (Qiagen), following the recommendations established by the manufacturer.

### DNA quantification, library construction and sequencing

The genomic DNA extracted from each batch of species was quantified using the Qubit 2.0 fluorometer (Life Technologies), with the Qubit dsDNA Hs Assay kit (Invitrogen), following the manufacturer instructions. The genomic libraries were constructed using the Ion Xpress Plus Fragment Library Kit (Thermo Fischer Scientific) in the AB Library Builder system (Life Technologies). In this step, the 200 bp library fragments were individually linked to barcode sequences for sample identification, using the Ion Xpress Barcode DNA Adaptors 1–32 Kit (Thermo Fischer Scientific). The prepared genomic libraries were subjected to a size selection (200 bp) by electrophoresis on 2% agarose gel, using the E-Gel SizeSelect (Invitrogen), aiming at the removal of short sequences, and then were quantified by *qPCR* on LightCycler 480 (Roche), using the Ion Library TaqMan quantitation kit (Thermo Fischer Scientific). Subsequently, the quantified fragments were amplified, through the *emPCR*, using the Ion PGM Hi-Q View OT2 kit (Thermo Fischer Scientific), in the Ion OneTouch 2 system (Thermo Fischer Scientific). Finally, the libraries were enriched with the Ion Xpress template kit (Thermo Fischer Scientific), deposited in the Ion 318 Chip Kit v2 BC (Thermo Fischer Scientific), and sequenced in the Ion Torrent PGM system (Thermo Fischer Scientific), using Ion PGM HI-Q View Sequencing (Thermo Fischer Scientific), following the recommendations established by the manufacturer.

### Mitochondrial genome assembly

The output files (Raw data) corresponding to the sequencing reads for each of the species evaluated were subjected to genomic assembly procedures by the De Novo method, using the IDBA UD v.1.1.1^82^ and SPAdes v.3.10.1^83^. Then, the selection of the regions corresponding to the mitogenome of each species was performed using DIAMOND^84^, these regions were visualized using MEGAN6^85^. The contigs with match to mitogenome was comparing with the available information of the complete sequencing of *Hg. janthinomys* (GenBank ID: NC28025), and inspected manually using Geneious v.9.1.8^86^.

### Sequence analysis

The obtained sequences were annotated using the online tool *MITOchondrial genome annotation Server*^87^ (MITOS—https://mitos.bioinf.uni-leipzig.de/), which also provided the prediction of the secondary structures of the tRNAs. Geneious v.9.1.8^86^ was used to cure sequences manually, and graphical maps of the mitogenomes were generated using the online tool *GenomeVx*^88^ (https://wolfe.ucd.ie/GenomeVx/). The nucleotide content values for each sequence were obtained using MEGA X^89^. The composition bias based on the asymmetry values (Whole genome, concatenated PCGs, individualized PCGs, concatenated tRNAs, and concatenated rRNAs) was estimated using the formulas: AT-skew = (A% − T%) / (A% + T%), and GC-skew = (G% − C%)/(G% + C%)^90^. A sliding window of 200 bp in steps 25 bp was performed using DnaSP v.6.12.03^91^ to estimate the diversity of nucleotides between the sequences obtained in this study, together with the data already available for *Hg. janthinomys* (GenBank ID: NC28025). In the PCGs, the relative use of synonymous codons (RSCU) was estimated using MEGA X^89^, and the pairwise comparison of the proportions of non-synonymous (*d*_*N*_) and synonymous (*d*_*S*_) substitutions (*d*_*N*_*/d*_*S*_) were calculated using the DnaSP v.6.12.03^91^. In this last analysis, the reasons obtained are used to estimate whether the protein-coding genes are under negative selection (purification) (*d*_*N*_*/d*_*S*_ < 1), positive selection (adaptive) (*d*_*N*_*/d*_*S*_ > 1), or neutral evolution.

### Phylogenetic analysis

The sequences containing the 13 PCGs of 27 mitogenomes (including the four mitogenomes sequenced in this study, and 23 mitogenomes available in the GenBank database—Supplementary Table 2) were extracted together using Geneious v.9.1.8^86^, and were aligned using the MAFFT algorithm^92^, being grouped into a single reading file. The nucleotide distances between the sequences used were obtained based on the alignment performed, using the Maximum Composite Likelihood model to build the distance matrix in the MEGA X^89^. The best nucleotide replacement model for phylogeny reconstruction was defined under the Akaike information criterion (AIC), using jModelTest v.2.1.7^93^. Bayesian inference was performed using MrBayes v.3.2.7a^94^ in two independent and simultaneous runs, with four chains each (three hot chains and one cold chain), established for 5,000,000 generations with a sampling of trees every 1000 steps, with relative burning of 25%. The Maximum Likelihood analysis was performed using RaxML v.8.2.11^95^, with bootstrapping values defined for 1,000 repetitions. The obtained topologies were visualized in FigTree v.1.4.4^96^.

## Supplementary information

Supplementary file 1.

## Data Availability

All data generated during this study are available as tables and figures included in this published article and its Supplementary Information files. The GenBank database accession numbers for the four mitochondrial genomes sequenced in this study are MN531846 (*Hg. albomaculatus*), MN531847 (*Hg. leucocelaenus*), MN531848 (*Hg. spegazzinii*), and MN531849 (*Hg. tropicalis*).
