# Supplementary material for: Mitochondrial genome sequencing and phylogeny of Haemagogus albomaculatus, Haemagogus leucocelaenus, Haemagogus spegazzinii, and Haemagogus tropicalis (Diptera: Culicidae)
Source: Sci Rep. 2020 Oct 12;10:16948. doi: 10.1038/s41598-020-73790-x (PMC7550346; doi:10.1038/s41598-020-73790-x)
Supplement: Supplementary file 1 [file 41598_2020_73790_MOESM1_ESM.pdf]

Supplementary information of

Sequencing mitochondrial genome and phylogenetic considerations of *Haemagogus albomaculatus, Haemagogus leucocelaenus, Haemagogus spegazzinii,* and *Haemagogus tropicalis* (Diptera: Culicidae)

Fábio Silva da Silva, Ana Cecília Ribeiro Cruz, Daniele Barbosa de Almeida Medeiros, Sandro Patroca da Silva, Márcio Roberto Teixeira Nunes, Lívia Carício Martins, Jennifer Chiang Oliveira, Poliana da Silva Lemos, Gabriel Muricy Cunha, Renato Freitas de Araujo, Joaquim Pinto Nunes Neto

Supplementary Table 1. Collection information of the analyzed *Haemagogus* species.

Supplementary Table 2. List of taxa used in the phylogenetic analyzes of this study.

Supplementary Table 3. Organization of *Hg. albomaculatus* mitogenome.

Supplementary Table 4. Organization of *Hg. leucocelaenus* mitogenome.

Supplementary Table 5. Organization of *Hg. spegazzinii* mitogenome.

Supplementary Table 6. Organization of *Hg. tropicalis* mitogenome.

Supplementary Table 7. Composition and skewness of sequenced *Haemagogus* mitogenomes. Composition and skewness in whole mitogenomes and concatenated PCGs, tRNAs, and rRNAs (**A**). AT% content in PCGs codons positions (**B**).

Supplementary Table 8. Relative synonymous codon usage (RSCU) in sequenced *Haemagogus* mitogenomes.

Supplementary Table 9. Matrix of nucleotide distances between the taxa used in this study, built by Maximum Composite Likelihood.

Supplementary Figure 1. Predicted secundary structure of tRNAs of *Hg. albomaculatus.*

Supplementary Figure 2. Predicted secundary structure of tRNAs of *Hg. leucocelaenus.*

Supplementary Figure 3. Predicted secundary structure of tRNAs of *Hg. spegazzinii.*

Supplementary Figure 4. Predicted secundary structure of tRNAs of *Hg. tropicalis.*

Supplementary Figure 5. Phylogenetic reconstruction by the Bayesian Inference method, based on the concatenation of the 13 PCGs of the *Haemagogus* species sequenced in this study and 23 other taxa with data available on GenBank database. The subsequent Bayesian probabilities (BP) are shown on the left, in each node. The complementary lines dotted in red indicate the newly-sequenced species.

Supplementary Figure 6. Phylogenetic reconstruction by the Maximum Likelihood methods, based on the concatenation of the 13 PCGs of the *Haemagogus* species sequenced in this study and 23 other taxa with data available on GenBank database. The values of support for bootstrapping (BPP) for 1,000 replicates are shown on the left, in each node. The complementary lines dotted in red indicate the newly-sequenced species.

Supplementary Figure 7. Phylogenetic reconstruction by the Bayesian Inference method, based on the concatenation of the genes *ATP8, COI, ND5,* and *ND6* of the *Haemagogus* species sequenced in this study and 03 other taxa with data available on GenBank database. The subsequent Bayesian probabilities (BP) are shown on the left, in each node. The complementary lines dotted in red indicate the newly-sequenced species.

Supplementary Figure 8. Phylogenetic reconstruction by the Maximum Likelihood methods, based on the concatenation of the genes *ATP8, COI, ND5,* and *ND6* of the *Haemagogus* species sequenced in this study and 03 other taxa with data available on GenBank database. The values of support for bootstrapping (BPP) for 1,000 replicates are shown on the left, in each node. The complementary lines dotted in red indicate the newly-sequenced species.

Supplementary Table 1. Collection information of the analyzed *Haemagogus* species.

Species	ID Code	Collected	Used	Sex	Collection location	State	Geographic coordinates	Year
[1]*Hg. albomaculatus*	AR_795522	5	*	♀	Jaborandi	Bahia	S 13°58'92" W 44°53'36"	2013
[2]*Hg. leucocelaenus*	AR_847534	23	7	♀	Canaã dos Carajás	Pará	S 06°49'75" W 49°87'83"	2016
[1]*Hg. spegazzinii*	AR_843225	30	*	♀	Coribe	Bahia	S 13°82'94" W 44°44'92"	2016
[3]*Hg. tropicalis*	AR_854398	6	4	♀	Ilha do Combu	Pará	S 01°51'97" W 48°49'28"	2018

[1]Authorization number for collection: Not applicable.

[2]Authorization number for collection: MMA/ICMBio/Carajás National Forest/Direct authorization nº 021/2018

[3]Authorization number for collection: MMA/ICMBio/SISBIO authorization nº 60595-1

* Macerated samples gently provided for total DNA extraction, after performing routine viral isolation procedures.

Supplementary Table 2. List of taxa used in the phylogenetic analyzes of this study.

Species used and taxonomic information	Size (bp)	GenBank ID	Reference
**Culicidae Family**			
**Culicinae Subfamily**			
**Aedini Tribe**			
***Haemagogus* Genus**			
***Conopstegus* Subgenus**			
Haemagogus leucocelaenus	15056	MN531847	This study
***Haemagogus* Subgenus**			
**Albomaculatus Section**			
Haemagogus albomaculatus	15097	MN531846	This study
Haemagogus janthinomys	15698	NC_028025	Lemos *et al.*[1]
Haemagogus spegazzinii	15081	MN531848	This study
**Tropicalis Section**			
Haemagogus tropicalis	14917	MN531849	This study
***Aedes* Genus**			
***Stegomyia* Subgenus**			
Aedes aegypti	16790	NC_035159	Matthews *et al.*[2]
Aedes albopictus	16665	NC_006817	Direct Submission
***Psorophora* Genus**			
***Janthinosoma* Subgenus**			
Psorophora ferox	15345	MK575485	Direct Submission
***Psorophora* Subgenus**			
Psorophora saeva	15049	NC_044658	Direct Submission
**Culicini Tribe**			
***Culex* Genus**			
***Culex* Subgenus**			
Culex camposi	15570	NC_036008	Demari-Silva *et al.*[3]
Culex coronator	15576	NC_036006	Demari-Silva *et al.*[3]
Culex pipiens pipiens	14856	NC_015079	Direct Submission
Culex quinquefasciatus	15587	NC_014574	Behura *et al.*[4]
***Lutzia* Genus**			
***Metalutzia* Subgenus**			
Lutzia fuscanus	15803	MH316118	Sun *et al.*[5]
Lutzia halifaxii	15744	MH316119	Sun *et al.*[5]
**Sabethini Tribe**			
***Sabethes* Genus**			
***Peytonulus* Subgenus**			
Sabethes undosus	15334	NC_044660	Lorenz *et al.*[6]
***Sabethes* Subgenus**			

Sabethes belisarioi	15911	NC_037498	Aragão *et al.*[7]
***Sabethoides* Subgenus**			
Sabethes chloropterus	15609	NC_037499	Aragão *et al.*[7]
Sabethes glaucodaemon	15620	NC_037500	Aragão *et al.*[7]
**Anophelinae Subfamily**			
***Anopheles* Genus**			
***Anopheles* Subgenus**			
Anopheles quadrimaculatus	15455	NC_000875	Mitchell *et al.*[8]
***Cellia* Subgenus**			
Anopheles gambiae	15363	NC_002084	Beard *et al.*[9]
***Kerteszia* Subgenus**			
Anopheles bellator	15668	NC_030249	Oliveira *et al.*[10]
Anopheles cruzii	15449	NC_024740	Direct Submission
Anopheles homunculus	15739	NC_030248	Oliveira *et al.*[10]
***Nyssorhynchus* Subgenus**			
Anopheles albitarsis	15413	NC_020662	Krzywinski *et al.*[11]
Anopheles darlingi	15386	NC_014275	Moreno *et al.*[12]
**Dixidae Family (Outgroup)**			
Dixella aestivalis	16465	NC_029354	Briscoe *et al.*[13]

References

1. Lemos, P. S. *et al.* Characterization of mitochondrial genome of *Haemagogus janthinomys* (Diptera: Culicidae). *Mitochondrial DNA Part A DNA Mapping, Seq. Anal.* **28**, 50–51 (2017).
2. Matthews, B. J. *et al.* Improved reference genome of *Aedes aegypti* informs arbovirus vector control. *Nature* **563**, 501–507 (2018).
3. Demari-Silva, B. *et al.* Mitochondrial genomes and comparative analyses of *Culex camposi*, *Culex coronator*, *Culex usquatus* and *Culex usquatissimus* (Diptera:Culicidae), members of the coronator group. *BMC Genomics* **16**, 831 (2015).
4. Behura, S. K. *et al.* Complete sequences of mitochondria genomes of *Aedes aegypti* and *Culex quinquefasciatus* and comparative analysis of mitochondrial dna fragments inserted in the nuclear genomes. *Insect Biochem. Mol. Biol.* **41**, 770–777 (2011).
5. Sun, L. *et al.* The complete mt genomes of *Lutzia halifaxia*, *Lt. fuscanus* and *Culex*

pallidothorax (Diptera: Culicidae) and comparative analysis of 16 *Culex* and *Lutzia* mt genome sequences. *Parasites and Vectors* **12**, 368 (2019).

6. Lorenz, C., Alves, J. M. P., Foster, P. G., Sallum, M. A. M. & Suesdek, L. First record of translocation in Culicidae (Diptera) mitogenomes: Evidence from the tribe Sabethini. *BMC Genomics* **20**, 721 (2019).
7. Aragão, A. O. *et al.* Description and phylogeny of the mitochondrial genome of *Sabethes chloropterus*, *Sabethes glaucodaemon* and *Sabethes belisarioi* (Diptera: Culicidae). *Genomics* **111**, 607–611 (2019).
8. Mitchell, S. E., Cockburn, A. F. & Seawright, J. A. The mitochondrial genome of *Anopheles quadrimaculatus* species A: Complete nucleotide sequence and gene organization. *Genome* **36**, 1058–1073 (1993).
9. Beard, C. B., Hamm, D. M. & Collins, F. H. The mitochondrial genome of the mosquito *Anopheles gambiae*: DNA sequence, genome organization, and comparisons with mitochondrial sequences of other insects. *Insect Mol. Biol.* **2**, 103–124 (1993).
10. Oliveira, T. M. P. *et al.* Mitochondrial Genomes of *Anopheles* (*Kerteszia*) (Diptera: Culicidae) from the Atlantic Forest, Brazil. *J. Med. Entomol.* **53**, 790–797 (2016).
11. Krzywinski, J. *et al.* Analysis of the evolutionary forces shaping mitochondrial genomes of a Neotropical malaria vector complex. *Mol. Phylogenet. Evol.* **58**, 469–477 (2011).
12. Moreno, M. *et al.* Complete mtDNA genomes of *Anopheles darlingi* and an approach to anopheline divergence time. *Malar. J.* **9**, 127 (2010).
13. Briscoe, A. G., Sivell, D. & Harbach, R. E. The complete mitochondrial genome of *Dixella aestivalis* (Diptera: Nematocera: Dixidae). *Mitochondrial DNA Part A DNA Mapping, Seq. Anal.* **28**, 83–84 (2017).

Supplementary Table 3. Organization of *Hg. albomaculatus* mitogenome.

Haemagogus albomaculatus										
**Gene**	**Strand**	**Position**		**Size (pb)**	**IGN**	**Anticodon**	**Start/Stop Codon**	**AT%**	**AT-skew**	**GC-skew**
		**Start**	**End**							
Ile (I)	J	1	68	68	0	GAU		75.0	0.0588	0.1765
Gln (Q)	N	66	134	69	-3	UUG		81.2	-0.0714	0.3846
Met (M)	J	134	202	69	-1	CAU		72.5	-0.0400	-0.1579
ND2	J	224	1186	963	21		ATA/TAA	83.4	-0.1059	-0.1375
Trp (W)	J	1234	1302	69	47	UCA		76.8	0.0943	-0.1250
Cys (C)	N	1302	1368	67	-1	GCA		83.6	0.0714	0.0909
Tyr (Y)	N	1369	1436	68	0	GUA		79.4	0.0741	0.4286
COI	J	1441	2949	1509	4		ATT/TAA	71.4	-0.1484	-0.0116
Leu (L2)	J	2972	3038	67	22	UAA		77.6	-0.0385	0.2000
COII	J	3040	3714	675	1		ATG/TAA	77.5	-0.1205	-0.0526
Lys (K)	J	3725	3795	71	10	CUU		73.2	0.0769	-0.0526
Asp (D)	J	3805	3874	70	9	GUC		87.1	0.0820	-0.1111
ATP8	J	3875	4033	159	0		ATT/TAA	84.9	-0.0963	-0.4167
ATP6	J	4030	4701	672	-4		TTG/TAA	76.5	-0.1206	-0.1519
COIII	J	4714	5499	786	12		ATG/TAA	73.3	-0.1597	-0.0095
Gly (G)	J	5502	5568	67	2	UCC		80.6	0.0370	0.0769
ND3	J	5587	5913	327	18		ATA/TAA	79.8	-0.1648	-0.0303
Arg (R)	J	5924	5988	65	10	UCG		67.7	-0.1364	0.0476
Ala (A)	J	5993	6059	67	4	UGC		85.1	-0.0526	0.2000
Asn (N)	J	6061	6130	70	1	GUU		78.6	0.0545	0.0667
Ser (S1)	N	6129	6195	67	-2	GCU		76.1	-0.0980	0.0000
Glu (E)	J	6212	6277	66	16	UUC		90.9	0.0333	-0.3333
Phe (F)	N	6292	6358	67	14	GAA		80.6	-0.0370	0.5385
ND5	N	6379	8067	1689	20		ATC/TAA	80.3	-0.1466	0.2530
His (H)	N	8107	8171	65	39	GUG		81.5	-0.1321	0.5000
ND4	N	8188	9522	1335	16		ATT/TAA	80.2	-0.1821	0.2727
ND4L	N	9519	9797	279	-4		ATC/TAA	83.9	-0.2308	0.3333
Thr (T)	J	9815	9879	65	17	UGU		87.7	0.0526	0.0000
Pro (P)	N	9880	9946	67	0	UGG		77.6	0.0769	0.4667
ND6	J	9952	10455	504	5		ATA/TAA	86.9	-0.1324	-0.0909
CytB	J	10467	11573	1107	11		ATT/TAA	75.7	-0.1599	-0.0260
Ser (S2)	J	11602	11669	68	28	UGA		82.4	0.0714	0.1667
ND1	N	11705	12616	912	35		ATA/TAA	78.0	-0.2405	0.2736
Leu (L1)	N	12647	12715	69	30	UAG		85.5	-0.0508	0.4000
rRNA 16S	N	12693	14052	1360	-23			83.2	-0.0618	0.2982
Val (V)	N	14055	14126	72	2	UAC		80.6	0.0690	0.1429
rRNA 12S	N	14126	14918	793	-1			81.6	-0.0325	0.2466
*	-	14919	15097	179	-	-	-	-	-	-

Supplementary Table 4. Organization of *Hg. leucocelaenus* mitogenome.

Haemagogus leucocelaenus										
**Gene**	**Strand**	**Position**		**Size (pb)**	**IGN**	**Anticodon**	**Start/Stop Codon**	**AT%**	**AT-skew**	**GC-skew**
		**Start**	**End**							
Ile (I)	J	1	67	67	0	GAU		74.6	0.0000	0.1765
Gln (Q)	N	65	133	69	-3	UUG		82.6	-0.0877	0.3333
Met (M)	J	133	201	69	-1	CAU		71.0	-0.1020	-0.2000
ND2	J	223	1209	987	21		ATA/TAA	83.5	-0.1238	-0.1534
Trp (W)	J	1235	1303	69	25	UCA		81.2	0.0357	-0.0769
Cys (C)	N	1303	1369	67	-1	GCA		83.6	0.1071	0.0909
Tyr (Y)	N	1370	1436	67	0	GUA		80.6	0.0741	0.3846
COI	J	1441	2949	1509	4		ATT/TAA	71.2	-0.1312	-0.0369
Leu (L2)	J	2972	3038	67	22	UAA		77.6	0.0000	0.2000
COII	J	3040	3720	681	1		ATG/TAA	75.6	-0.0796	-0.1807
Lys (K)	J	3725	3795	71	4	CUU		74.6	0.0943	0.0000
Asp (D)	J	3812	3880	69	16	GUC		87.0	0.0667	-0.1111
ATP8	J	3882	4040	159	1		ATT/TAA	82.4	-0.0382	-0.4286
ATP6	J	4037	4708	672	-4		TTG/TAA	74.9	-0.1213	-0.2189
COIII	J	4721	5506	786	12		ATG/TAA	72.0	-0.1378	-0.0818
Gly (G)	J	5509	5575	67	2	UCC		79.1	0.0566	0.0000
ND3	J	5594	5920	327	18		ATA/TAA	79.8	-0.1648	-0.0303
Arg (R)	J	5931	5994	64	10	UCG		70.3	-0.0667	-0.0526
Ala (A)	J	5999	6065	67	4	UGC		79.1	-0.0189	0.2857
Asn (N)	J	6067	6136	70	1	GUU		80.0	0.1429	0.1429
Ser (S1)	N	6135	6201	67	-2	GCU		73.1	-0.0612	0.0000
Glu (E)	J	6210	6275	66	8	UUC		90.9	0.0333	-0.3333
Phe (F)	N	6278	6344	67	2	GAA		83.6	0.0000	0.6364
ND5	N	6369	8054	1686	24		ATC/TAA	79.7	-0.1780	0.3178
His (H)	N	8094	8158	65	39	GUG		81.5	-0.0566	0.5000
ND4	N	8178	9509	1332	19		ATT/TAA	79.7	-0.2064	0.3284
ND4L	N	9506	9784	279	-4		ATC/TAA	81.4	-0.2863	0.4615
Thr (T)	J	9802	9866	65	17	UGU		87.7	0.0526	0.0000
Pro (P)	N	9867	9933	67	0	UGG		77.6	0.0385	0.4667
ND6	J	9939	10442	504	5		ATA/TAA	84.7	-0.1101	-0.3247
CytB	J	10475	11560	1086	32		ATT/TAA	74.1	-0.1404	-0.0961
Ser (S2)	J	11590	11655	66	29	UGA		80.3	0.0943	0.0769
ND1	N	11682	12614	933	26		ATA/TAA	78.1	-0.2455	0.2941
Leu (L1)	N	12624	12691	68	9	UAG		85.3	0.0000	0.4000
rRNA 16S	N	12664	14028	1365	-28			82.9	-0.0583	0.2704
Val (V)	N	14027	14098	72	-2	UAC		80.6	0.0345	0.1429
rRNA 12S	N	14098	14889	792	-1			81.7	-0.0386	0.2552
*	-	14890	15056	167	-	-	-	-	-	-

Supplementary Table 5. Organization of *Hg. spegazzinii* mitogenome.

Haemagogus spegazzinii										
**Gene**	**Strand**	**Position**		**Size (pb)**	**IGN**	**Anticodon**	**Start/Stop Codon**	**AT%**	**AT-skew**	**GC-skew**
		**Start**	**End**							
Ile (I)	J	1	68	68	0	GAU		75.0	0.0588	0.1765
Gln (Q)	N	66	134	69	-3	UUG		81.2	-0.0357	0.3846
Met (M)	J	134	202	69	-1	CAU		72.5	-0.0400	-0.1579
ND2	J	224	1210	987	21		ATA/TAA	84.1	-0.1084	-0.0446
Trp (W)	J	1234	1302	69	23	UCA		78.3	0.0741	-0.0667
Cys (C)	N	1302	1368	67	-1	GCA		80.6	0.0741	0.2308
Tyr (Y)	N	1369	1436	68	0	GUA		79.4	0.0741	0.4286
COI	J	1441	2949	1509	4		ATT/TAA	70.8	-0.1581	-0.0136
Leu (L2)	J	2972	3038	67	22	UAA		76.1	-0.0588	0.1250
COII	J	3040	3714	675	1		ATG/TAA	76.3	-0.1068	-0.0875
Lys (K)	J	3725	3795	71	10	CUU		73.2	0.0769	-0.0526
Asp (D)	J	3810	3878	69	14	GUC		87.0	0.0667	-0.1111
ATP8	J	3879	4037	159	0		ATT/TAA	84.9	-0.1259	-0.4167
ATP6	J	4034	4705	672	-4		TTG/TAA	76.6	-0.1223	-0.1592
COIII	J	4718	5503	786	12		ATG/TAA	72.9	-0.1658	0.0047
Gly (G)	J	5506	5572	67	2	UCC		80.6	0.0000	0.0769
ND3	J	5588	5917	330	15		ATA/TAA	80.9	-0.1760	0.0159
Arg (R)	J	5928	5991	64	10	UCG		68.8	-0.0455	0.0000
Ala (A)	J	5996	6062	67	4	UGC		82.1	-0.0182	0.0000
Asn (N)	J	6064	6132	69	1	GUU		78.3	0.1481	0.0667
Ser (S1)	N	6131	6197	67	-2	GCU		76.1	-0.0980	0.0000
Glu (E)	J	6217	6282	66	19	UUC		90.9	0.0333	-0.3333
Phe (F)	N	6290	6356	67	7	GAA		80.6	0.0370	0.3846
ND5	N	6377	8065	1689	20		ATC/TAA	79.0	-0.1416	0.2486
His (H)	N	8105	8169	65	39	GUG		81.5	-0.0943	0.5000
ND4	N	8186	9520	1335	16		ATT/TAA	80.4	-0.1892	0.2824
ND4L	N	9517	9795	279	-4		ATC/TAA	83.2	-0.2500	0.2766
Thr (T)	J	9813	9877	65	17	UGU		87.7	0.0877	0.0000
Pro (P)	N	9878	9944	67	0	UGG		77.6	0.0769	0.4667
ND6	J	9950	10453	504	5		ATA/TAA	86.7	-0.0709	-0.1642
CytB	J	10465	11571	1107	11		ATT/TAA	74.6	-0.1598	-0.0036
Ser (S2)	J	11600	11665	66	28	UGA		81.8	0.0741	0.1667
ND1	N	11711	12628	918	45		ATA/TAA	79.1	-0.2204	0.2500
Leu (L1)	N	12638	12705	68	9	UAG		85.3	-0.0345	0.4000
rRNA 16S	N	12672	14029	1358	-34			83.1	-0.0487	0.2926
Val (V)	N	14042	14113	72	12	UAC		80.6	0.0690	0.1429
rRNA 12S	N	14113	14906	794	-1			81.2	-0.0295	0.2617
*	-	14907	15081	175	-	-	-	-	-	-

Supplementary Table 6. Organization of *Hg. tropicalis* mitogenome.

Haemagogus tropicalis										
**Gene**	**Strand**	**Position**		**Size (pb)**	**IGN**	**Anticodon**	**Start/Stop Codon**	**AT%**	**AT-skew**	**GC-skew**
		**Start**	**End**							
Ile (I)	J	1	67	67	0	GAU		74.6	0.0800	0.1765
Gln (Q)	N	68	136	69	0	UUG		82.6	-0.0877	0.3333
Met (M)	J	136	204	69	-1	CAU		71.0	-0.0612	-0.2000
ND2	J	226	1212	987	21		ATA/TAA	84.2	-0.1047	-0.0769
Trp (W)	J	1236	1304	69	23	UCA		82.6	0.1228	-0.1667
Cys (C)	N	1304	1370	67	-1	GCA		83.6	0.0357	0.0909
Tyr (Y)	N	1371	1438	68	0	GUA		80.9	0.0909	0.3846
COI	J	1443	2951	1509	4		ATT/TAA	72.2	-0.1111	-0.0190
Leu (L2)	J	2974	3040	67	22	UAA		79.1	-0.0566	0.1429
COII	J	3043	3714	672	2		ATT/TAA	76.0	-0.0646	-0.1429
Lys (K)	J	3728	3798	71	13	CUU		74.6	0.0943	0.0000
Asp (D)	J	3812	3880	69	13	GUC		87.0	0.1000	-0.1111
ATP8	J	3881	4039	159	0		ATT/TAA	83.0	-0.0758	-0.4815
ATP6	J	4036	4707	672	-4		TTG/TAA	76.6	-0.0990	-0.1847
COIII	J	4732	5505	774	24		ATG/TAA	73.8	-0.1454	-0.0246
Gly (G)	J	5508	5574	67	2	UCC		79.1	0.0566	0.0000
ND3	J	5581	5919	339	6		ATA/TAA	81.1	-0.1709	-0.0625
Arg (R)	J	5927	5990	64	7	UCG		71.9	-0.0870	0.0000
Ala (A)	J	5997	6063	67	6	UGC		85.1	-0.0175	0.2000
Asn (N)	J	6065	6134	70	1	GUU		78.6	0.0545	0.0667
Ser (S1)	N	6133	6199	67	-2	GCU		74.6	-0.1200	0.0588
Glu (E)	J	6228	6293	66	28	UUC		92.4	0.1148	-0.2000
Phe (F)	N	6292	6358	67	-2	GAA		86.6	0.0000	0.5556
ND5	N	6379	8061	1683	20		ATC/TAA	80.4	-0.1530	0.2606
His (H)	N	8107	8171	65	45	GUG		81.5	-0.0943	0.5000
ND4	N	8189	9523	1335	17		ATG/TAA	81.5	-0.1765	0.2227
ND4L	N	9520	9786	267	-4		ATG/TAA	83.1	-0.2613	0.4667
Thr (T)	J	9816	9880	65	29	UGU		87.7	0.0526	0.0000
Pro (P)	N	9881	9947	67	0	UGG		80.6	0.0000	0.5385
ND6	J	9956	10459	504	8		ATA/TAA	86.1	-0.1014	-0.2571
CytB	J	10471	11595	1125	11		ATG/TAA	75.1	-0.1266	-0.0500
Ser (S2)	J	11610	11675	66	14	UGA		81.8	0.0370	0.1667
ND1	N	11704	12615	912	28		ATA/TAA	78.2	-0.2482	0.2764
Leu (L1)	N	12646	12713	68	30	UAG		85.3	-0.0345	0.4000
rRNA 16S	N	12680	14036	1357	-34			83.4	-0.0495	0.2889
Val (V)	N	14050	14121	72	13	UAC		80.6	0.0345	0.1429
rRNA 12S	N	14121	14912	792	-1			82.2	-0.0138	0.2624
*	-	14913	14917	5	-	-	-	-	-	-

Supplementary Table 7. Composition and skewness of sequenced *Haemagogus* mitogenomes. Composition and skewness in whole mitogenomes and concatenated PCGs, tRNAs, and rRNAs (**A**). AT% content in PCGs codons positions (**B**).

A

Species	Size (bp)	A%	T%	C%	G%	AT%	AT-Skew	GC-Skew
**Whole mitogenome**								
Hg. albomaculatus	15097	40.0	39.5	11.8	8.7	79.5	0.0055	-0.1540
Hg. leucocelaenus	15056	40.0	38.8	12.7	8.5	78.8	0.0159	-0.2005
Hg. spegazzinii	15081	39.8	39.3	12.0	8.9	79.1	0.0059	-0.1455
Hg. tropicalis	14917	40.5	39.2	11.8	8.5	79.7	0.0160	-0.1637
Average	15038	40.1	39.2	12.1	8.6	79.3	0.0108	-0.1659
**PCGs**								
Hg. albomaculatus	10917	33.0	45.2	10.3	11.5	78.2	-0.1556	0.0589
Hg. leucocelaenus	10941	32.6	44.8	10.9	11.7	77.4	-0.1574	0.0364
Hg. spegazzinii	10950	33.0	44.9	10.3	11.7	77.9	-0.1534	0.0625
Hg. tropicalis	10938	33.7	44.8	10.4	11.2	78.4	-0.1419	0.0386
Average	10937	33.1	44.9	10.5	11.5	78.0	-0.1521	0.0491
**tRNAs**								
Hg. albomaculatus	1493	40.5	39.6	8.6	11.4	80.0	0.0109	0.1409
Hg. leucocelaenus	1486	40.9	39.2	8.6	11.3	80.1	0.0218	0.1351
Hg. spegazzinii	1487	40.9	38.9	8.8	11.4	79.8	0.0253	0.1296
Hg. tropicalis	1487	41.2	39.8	8.3	10.8	81.0	0.0166	0.1307
Average	1488	40.9	39.4	8.6	11.2	80.2	0.0187	0.1341
**rRNAs**								
Hg. albomaculatus	2153	39.2	43.4	6.3	11.1	82.6	-0.0512	0.2781
Hg. leucocelaenus	2157	39.1	43.3	6.4	11.1	82.5	-0.0512	0.2646
Hg. spegazzinii	2152	39.5	42.9	6.3	11.2	82.4	-0.0417	0.2804
Hg. tropicalis	2149	40.0	43.0	6.1	10.9	83.0	-0.0365	0.2787
Average	2153	39.4	43.2	6.3	11.1	82.6	-0.0451	0.2754

B

Species	AT% PCGs codons		
	1st	2nd	3rd
Hg. albomaculatus	71,2	67,4	96,1
Hg. leucocelaenus	71,0	67,6	93,5
Hg. spegazzinii	70,7	67,3	95,7
Hg. tropicalis	71,7	68,0	95,6
Average	71,2	67,6	95,2

Supplementary Table 8. Relative synonymous codon usage (RSCU) in sequenced *Haemagogus* mitogenomes.

Amino acid	Codons	*Hg. albomaculatus*		*Hg. leucocelaenus*		*Hg. spegazzinii*		*Hg. tropicalis*	
		Count	RSCU	Count	RSCU	Count	RSCU	Count	RSCU
Alanine (A)	GCU	100	2.58	93	2.45	99	2.51	75	2.11
	GCC	6	0.15	9	0.24	5	0.13	11	0.31
	GCA	47	1.21	46	1.21	50	1.27	54	1.52
	GCG	2	0.05	4	0.11	4	0.10	2	0.06
Arginine (R)	CGU	14	1.00	12	0.87	10	0.71	15	1.11
	CGC	0	0.00	1	0.07	1	0.07	3	0.22
	CGA	40	2.86	38	2.76	45	3.21	32	2.37
	CGG	2	0.14	4	0.29	0	0.00	4	0.30
Aspartate (D)	GAU	66	1.97	60	1.82	67	1.97	60	1.88
	GAC	1	0.03	6	0.18	1	0.03	4	0.13
Asparagine (N)	AAU	196	1.90	191	1.85	198	1.94	213	1.98
	AAC	10	0.10	15	0.15	6	0.06	2	0.02
Cysteine (C)	UGU	35	1.89	36	1.89	36	1.95	36	2.00
	UGC	2	0.11	2	0.11	1	0.05	0	0.00
Glutamine (Q)	CAA	70	1.92	73	1.95	73	1.97	72	1.95
	CAG	3	0.08	2	0.05	1	0.03	2	0.05
Glutamate (E)	GAA	77	1.97	74	1.90	77	1.97	76	1.95
	GAG	1	0.03	4	0.10	1	0.03	2	0.05
Glycine (G)	GGU	54	1.02	50	0.93	46	0.86	56	1.05
	GGC	2	0.04	2	0.04	2	0.04	3	0.06
	GGA	132	2.49	137	2.56	138	2.57	136	2.54
	GGG	24	0.45	25	0.47	29	0.54	19	0.36
Histidine (H)	CAU	71	1.82	61	1.61	68	1.74	68	1.79
	CAC	7	0.18	15	0.39	10	0.26	8	0.21
Isoleucine (I)	AUU	362	1.96	370	1.91	362	1.97	373	1.94
	AUC	8	0.04	17	0.09	6	0.03	11	0.06
Leucine (L) UUN	UUA	529	5.47	496	5.17	529	5.50	520	5.33
	UUG	12	0.12	25	0.26	7	0.07	12	0.12
Leucine (L) CUN	CUU	24	0.25	29	0.30	27	0.28	28	0.29
	CUC	0	0.00	1	0.01	1	0.01	3	0.03
	CUA	13	0.13	25	0.26	13	0.14	21	0.22
	CUG	2	0.02	0	0.00	0	0.00	1	0.01
Lysine (K)	AAA	87	1.91	83	1.82	79	1.74	88	1.87
	AAG	4	0.09	8	0.18	12	0.26	6	0.13
Methionine (M)	AUA	218	1.90	219	1.88	222	1.93	239	1.93
	AUG	11	0.10	14	0.12	8	0.07	9	0.07

**Phenylalanine (F)**	**UUU**	351	1.93	341	1.85	341	1.89	350	1.96
	**UUC**	12	0.07	28	0.15	19	0.11	8	0.04
**Proline (P)**	**CCU**	82	2.58	84	2.63	91	2.87	75	2.36
	**CCC**	13	0.41	8	0.25	7	0.22	12	0.38
	**CCA**	32	1.01	35	1.09	29	0.91	38	1.20
	**CCG**	0	0.00	1	0.03	0	0.00	2	0.06
**Serine (S) AGN**	**AGU**	45	1.15	41	1.05	45	1.13	39	0.99
	**AGC**	2	0.05	2	0.05	0	0.00	3	0.08
	**AGA**	64	1.63	65	1.66	68	1.71	67	1.70
	**AGG**	0	0.00	0	0.00	0	0.00	0	0.00
**Serine (S) UCN**	**UCU**	108	2.75	115	2.94	104	2.62	95	2.41
	**UCC**	3	0.08	5	0.13	9	0.23	9	0.23
	**UCA**	91	2.32	84	2.15	90	2.26	100	2.54
	**UCG**	1	0.03	1	0.03	2	0.05	2	0.05
**Threonine (T)**	**ACU**	93	2.00	99	2.19	92	2.02	87	1.94
	**ACC**	4	0.09	13	0.29	4	0.09	7	0.16
	**ACA**	88	1.89	68	1.50	86	1.89	85	1.90
	**ACG**	1	0.02	1	0.02	0	0.00	0	0.00
**Tryptophan (W)**	**UGA**	96	1.94	93	1.86	95	1.92	98	1.96
	**UGG**	3	0.06	7	0.14	4	0.08	2	0.04
**Tyrosine (Y)**	**UAU**	151	1.96	144	1.89	150	1.91	149	1.92
	**UAC**	3	0.04	8	0.11	7	0.09	6	0.08
**Valine (V)**	**GUU**	79	1.93	67	1.71	83	1.92	69	1.86
	**GUC**	2	0.05	5	0.13	6	0.14	6	0.16
	**GUA**	81	1.98	81	2.06	80	1.85	70	1.89
	**GUG**	2	0.05	4	0.10	4	0.09	3	0.08
**Total codons in use**		3639		3647		3650		3646	

Supplementary Table 9. Matrix of nucleotide distances between the taxa used in this study, built by Maximum Composite Likelihood.

		**1**	**2**	**3**	**4**	**5**	**6**	**7**	**8**	**9**	**10**	**11**	**12**	**13**	**14**	**15**	**16**	**17**	**18**	**19**	**20**	**21**	**22**	**23**	**24**	**25**	**26**
**1**	*Culex camposi* NC036008																										
**2**	*Culex coronator* NC036006	0,01																									
**3**	*Culex pipiens pipiens* NC015079	0,06	0,07																								
**4**	*Culex quinquefasciatus* NC014574	0,08	0,09	0,04																							
**5**	*Lutzia fuscanus* MH316118	0,08	0,08	0,09	0,10																						
**6**	*Lutzia halifaxii* MH316119	0,09	0,09	0,09	0,11	0,04																					
**7**	*Aedes aegypti* NC035159	0,14	0,14	0,14	0,10	0,14	0,14																				
**8**	*Aedes albopictus* NC006817	0,15	0,15	0,15	0,14	0,15	0,15	0,12																			
**9**	*Haemagogus albomaculatus* MN531846	0,13	0,13	0,13	0,13	0,13	0,13	0,12	0,13																		
**10**	*Haemagogus spegazzinii* MN531848	0,13	0,13	0,13	0,13	0,13	0,13	0,12	0,13	0,05																	
**11**	***Haemagogus janthinomys* NC028025**	0,13	0,13	0,13	0,12	0,13	0,13	0,12	0,12	**0,05**	**0,06**																
**12**	*Haemagogus leucocelaenus* MN531847	0,14	0,14	0,14	0,14	0,14	0,14	0,13	0,14	0,09	0,09	**0,09**															
**13**	*Haemagogus tropicalis* MN531849	0,14	0,14	0,14	0,14	0,14	0,14	0,14	0,14	0,10	0,10	**0,10**	0,11														
**14**	*Psorophora ferox* MK575485	0,12	0,12	0,13	0,13	0,13	0,13	0,13	0,14	0,12	0,12	0,12	0,13	0,14													
**15**	*Psorophora saeva* NC044658	0,13	0,13	0,13	0,13	0,13	0,13	0,13	0,14	0,11	0,12	0,12	0,13	0,13	0,09												
**16**	*Sabethes belisarioi* NC037498	0,16	0,16	0,17	0,17	0,16	0,16	0,18	0,17	0,15	0,15	0,15	0,16	0,16	0,17	0,16											
**17**	*Sabethes chloropterus* NC037499	0,18	0,18	0,18	0,18	0,18	0,18	0,19	0,19	0,17	0,17	0,17	0,18	0,18	0,18	0,18	0,13										
**18**	*Sabethes glaucodaemon* NC037500	0,17	0,17	0,17	0,17	0,17	0,17	0,17	0,17	0,15	0,15	0,15	0,16	0,16	0,17	0,16	0,11	0,10									
**19**	*Sabethes undosus* NC044660	0,17	0,18	0,18	0,18	0,17	0,17	0,18	0,18	0,16	0,16	0,16	0,17	0,17	0,17	0,17	0,12	0,12	0,11								
**20**	*Anopheles albitarsis* NC020662	0,17	0,17	0,17	0,17	0,17	0,18	0,18	0,18	0,17	0,17	0,17	0,18	0,18	0,17	0,18	0,20	0,21	0,19	0,20							
**21**	*Anopheles darlingi* NC014275	0,17	0,17	0,17	0,17	0,17	0,17	0,17	0,17	0,17	0,17	0,16	0,17	0,17	0,17	0,17	0,19	0,21	0,19	0,20	0,08						
**22**	*Anopheles gambiae* NC002084	0,16	0,17	0,17	0,17	0,17	0,17	0,17	0,18	0,17	0,17	0,17	0,18	0,18	0,17	0,17	0,19	0,21	0,19	0,20	0,13	0,12					
**23**	*Anopheles quadrimaculatus* NC000875	0,17	0,17	0,17	0,18	0,17	0,17	0,18	0,18	0,17	0,17	0,17	0,18	0,19	0,18	0,17	0,20	0,21	0,20	0,21	0,13	0,13	0,12				
**24**	*Anopheles bellator* NC030249	0,18	0,18	0,18	0,18	0,18	0,18	0,18	0,19	0,17	0,18	0,18	0,18	0,19	0,18	0,18	0,19	0,20	0,19	0,20	0,14	0,14	0,14	0,14			
**25**	*Anopheles cruzii* NC024740	0,17	0,18	0,18	0,18	0,18	0,18	0,18	0,18	0,17	0,17	0,17	0,18	0,18	0,18	0,18	0,19	0,20	0,19	0,20	0,14	0,13	0,14	0,14	0,07		
**26**	*Anopheles homunculus* NC030248	0,18	0,18	0,18	0,18	0,18	0,18	0,18	0,19	0,17	0,17	0,18	0,18	0,18	0,19	0,18	0,19	0,21	0,20	0,20	0,14	0,14	0,14	0,14	0,07	0,07	
**27**	***Dixella aestivalis* NC029354**	0,26	0,26	0,26	0,27	0,26	0,26	0,27	0,27	**0,25**	**0,26**	0,26	**0,26**	**0,26**	0,26	0,27	0,27	0,28	0,27	0,28	0,26	0,26	0,26	0,26	0,26	0,26	0,26

Taxon with the lowest mean nucleotide divergence compared to sequenced *Hg. spp*.
Taxon with the highest mean nucleotide divergence compared to sequenced *Hg. spp*.

Hg. albomaculatus

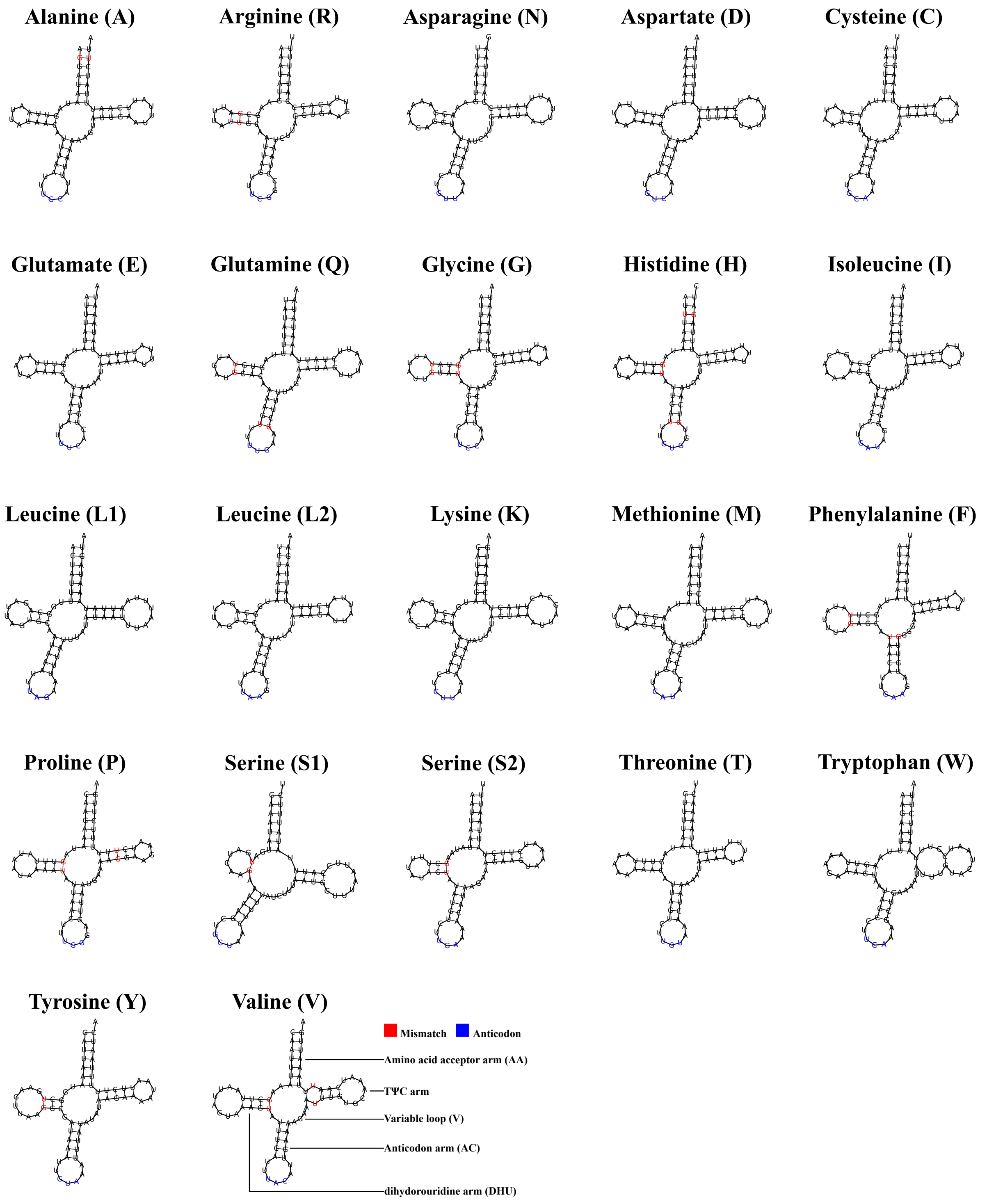

Supplementary Figure 1. Predicted secundary structure of tRNAs of *Hg. albomaculatus.*

Hg. leucocelaenus

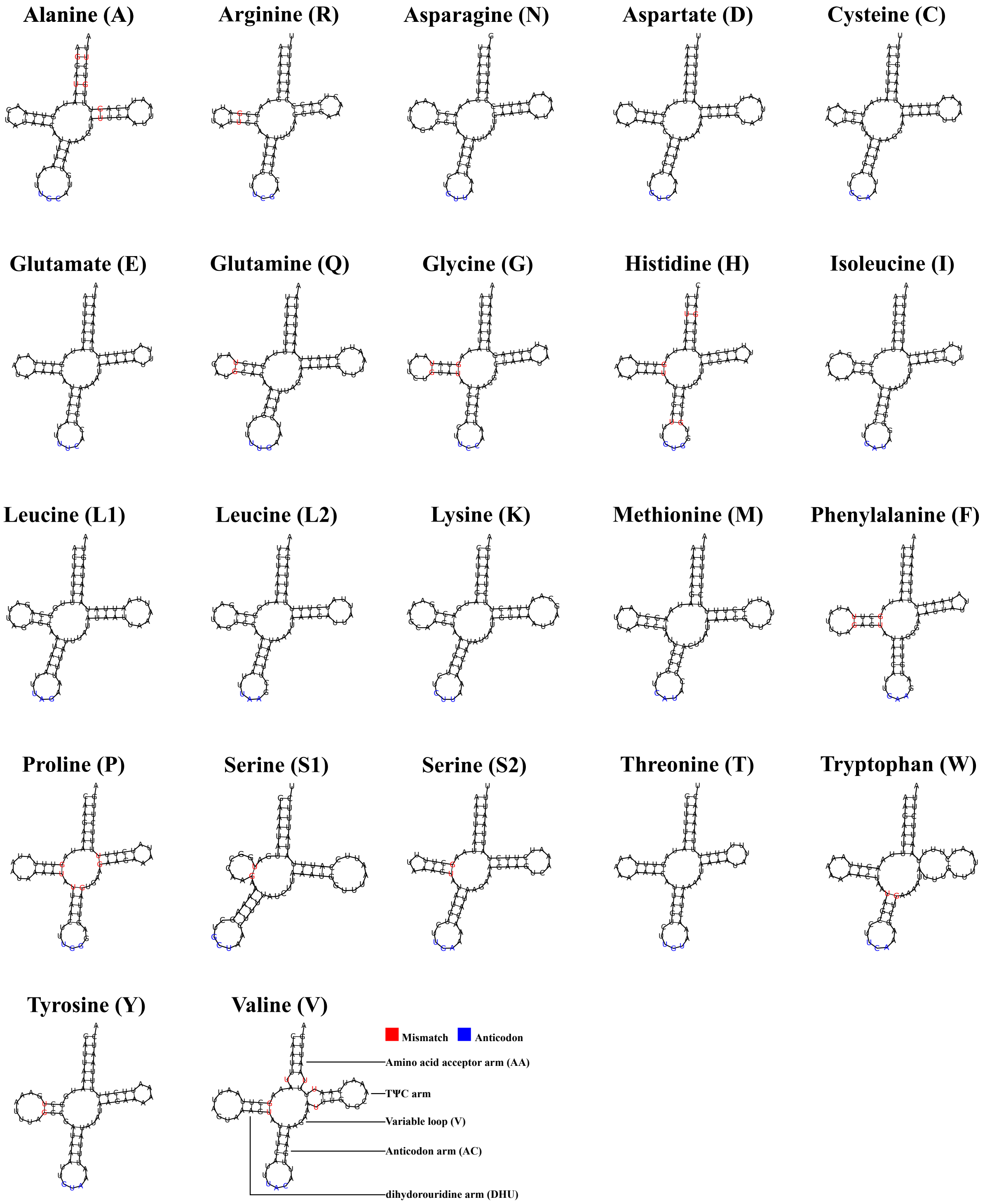

Supplementary Figure 2. Predicted secundary structure of tRNAs of *Hg. leucocelaenus.*

Hg. spegazzinii

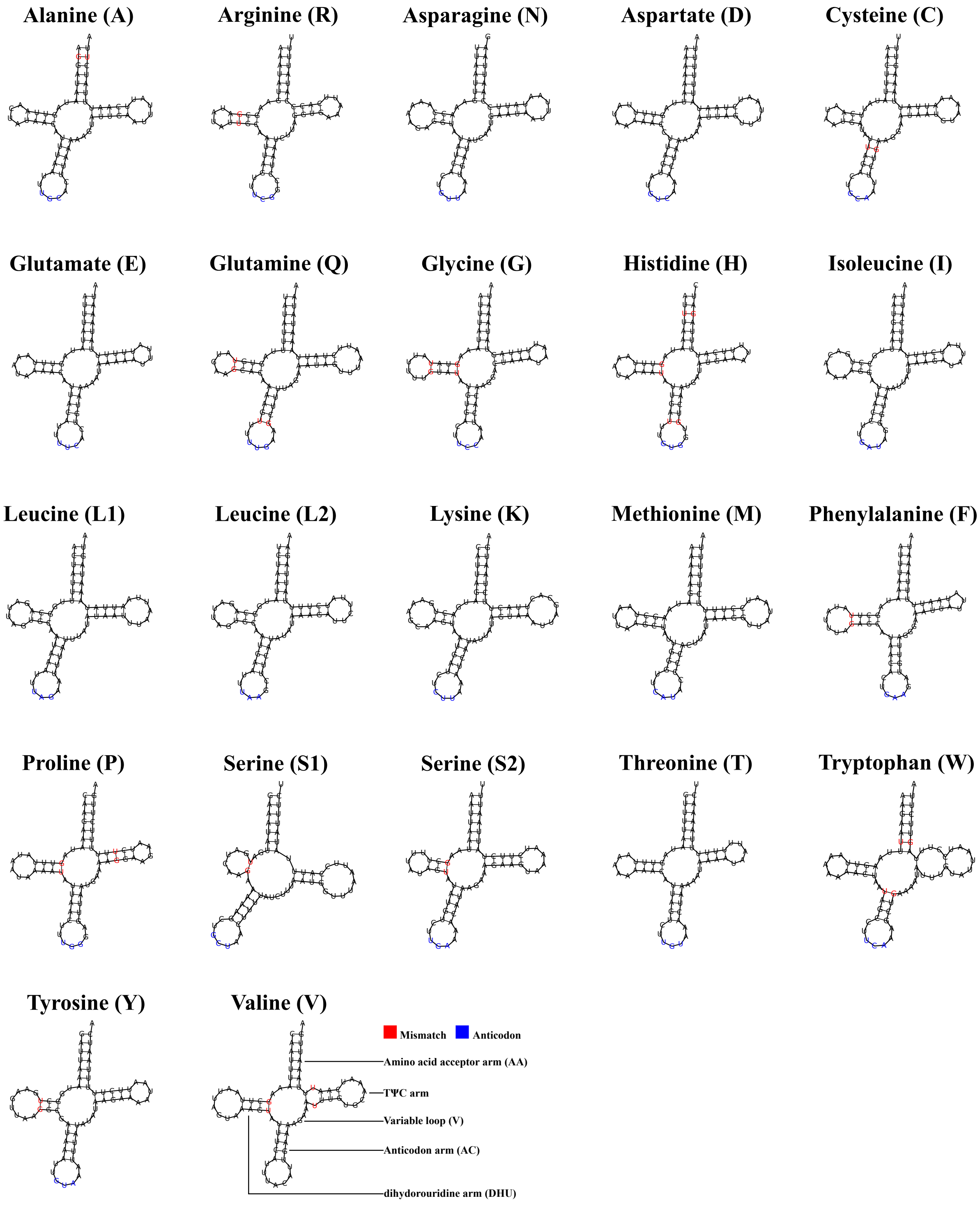

Supplementary Figure 3. Predicted secundary structure of tRNAs of *Hg. spegazzinii.*

Hg. tropicalis

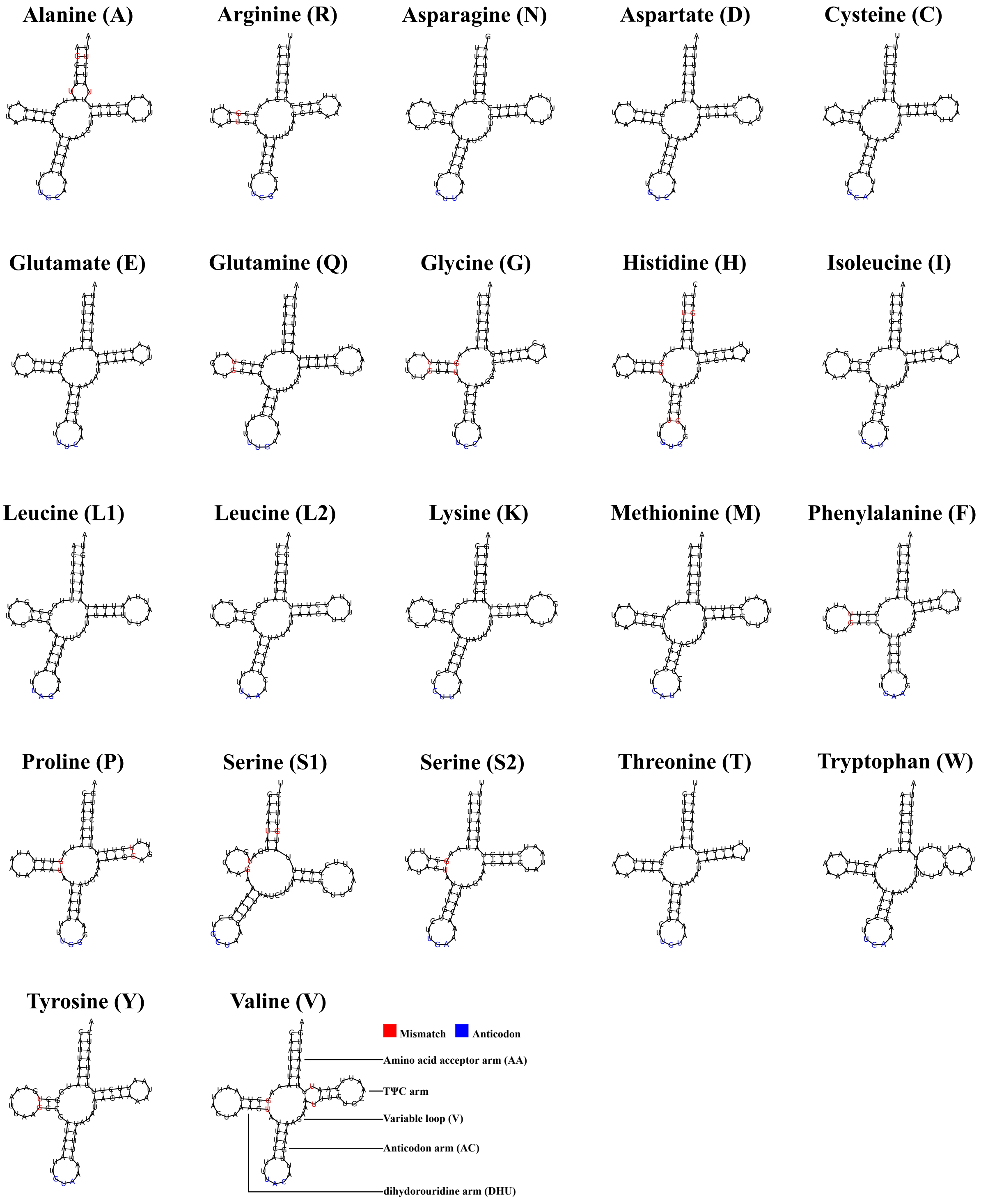

Supplementary Figure 4. Predicted secundary structure of tRNAs of *Hg. tropicalis.*

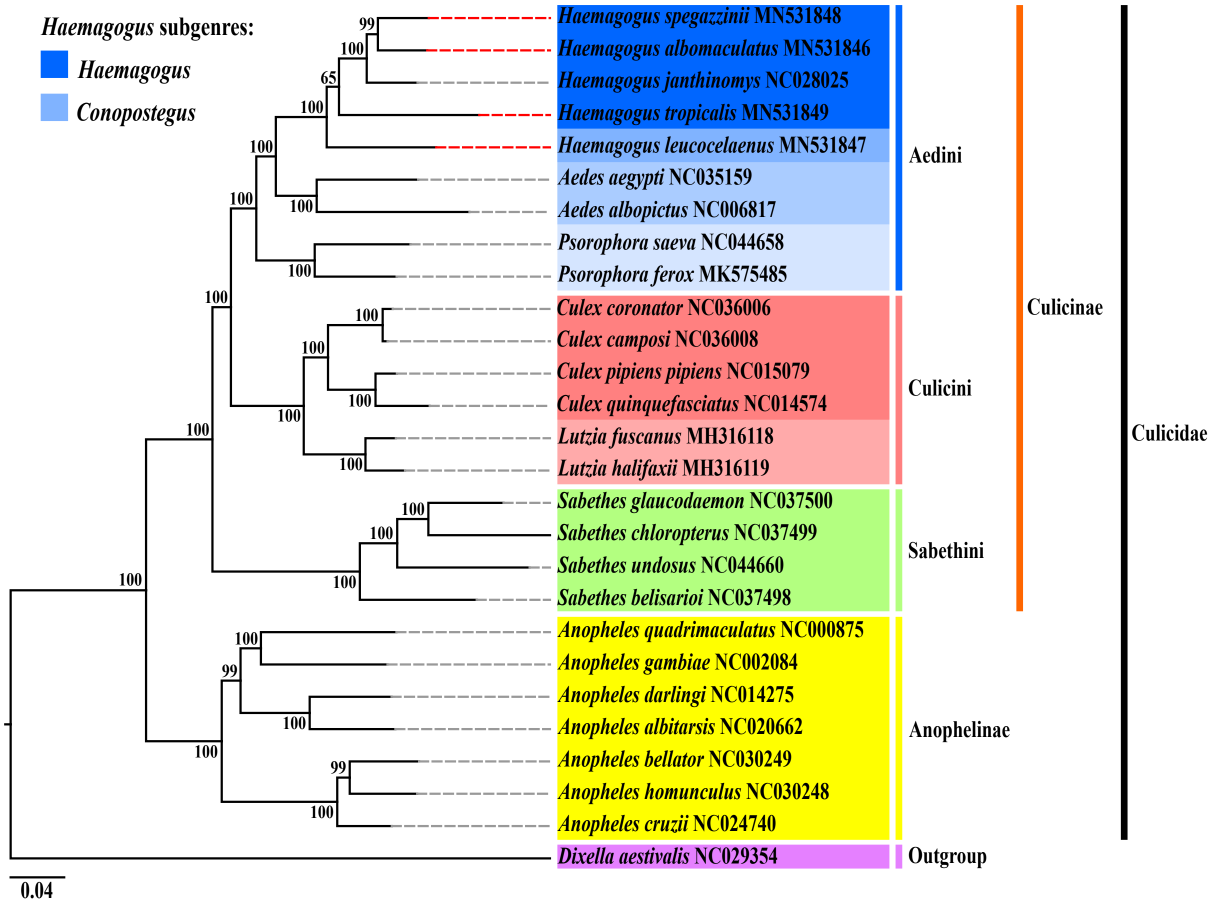

Supplementary Figure 5. Phylogenetic reconstruction by the Bayesian Inference method, based on the concatenation of the 13 PCGs of the *Haemagogus* species sequenced in this study and 23 other taxa with data available on GenBank database. The subsequent Bayesian probabilities (BP) are shown on the left, in each node. The complementary lines dotted in red indicate the newly-sequenced species.

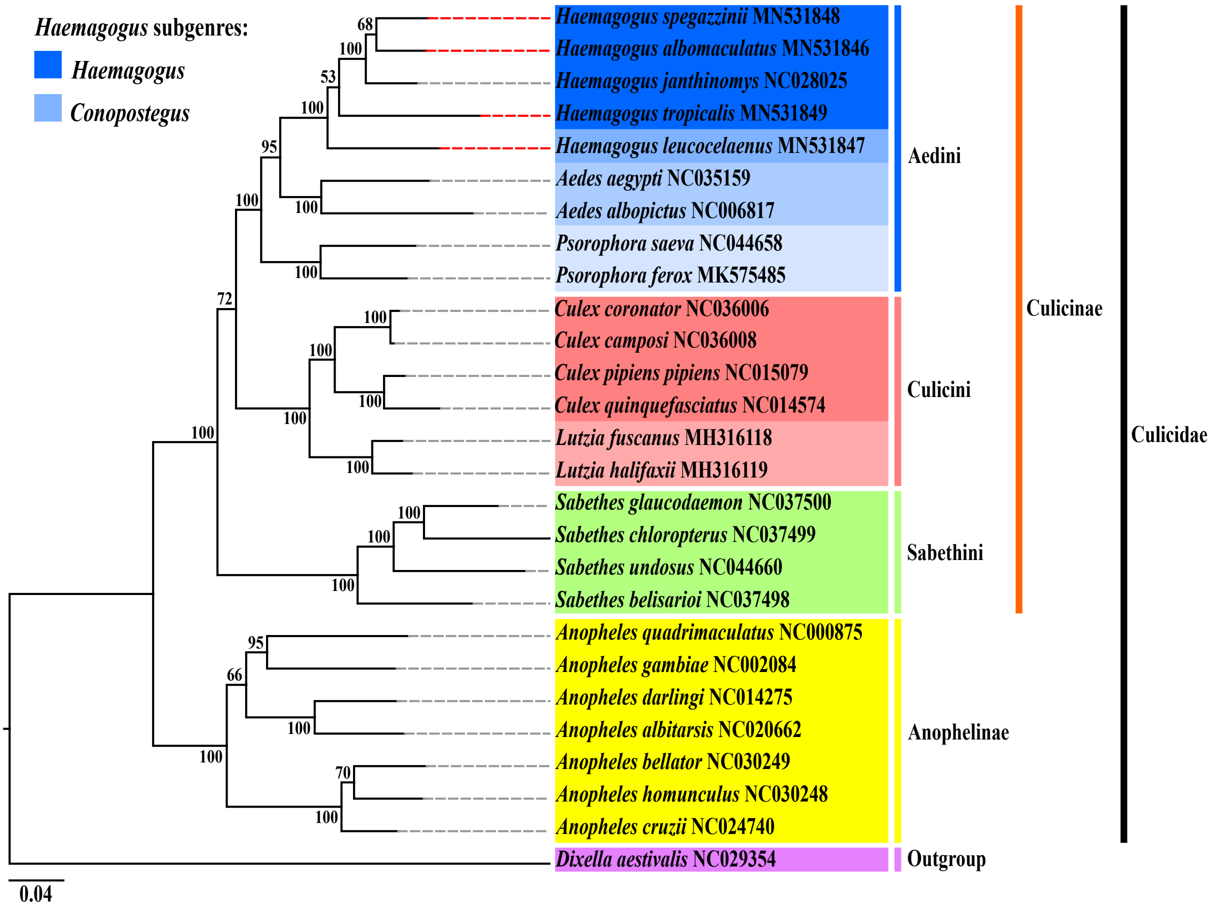

Supplementary Figure 6. Phylogenetic reconstruction by the Maximum Likelihood methods, based on the concatenation of the 13 PCGs of the *Haemagogus* species sequenced in this study and 23 other taxa with data available on GenBank database. The values of support for bootstrapping (BPP) for 1,000 replicates are shown on the left, in each node. The complementary lines dotted in red indicate the newly-sequenced species.

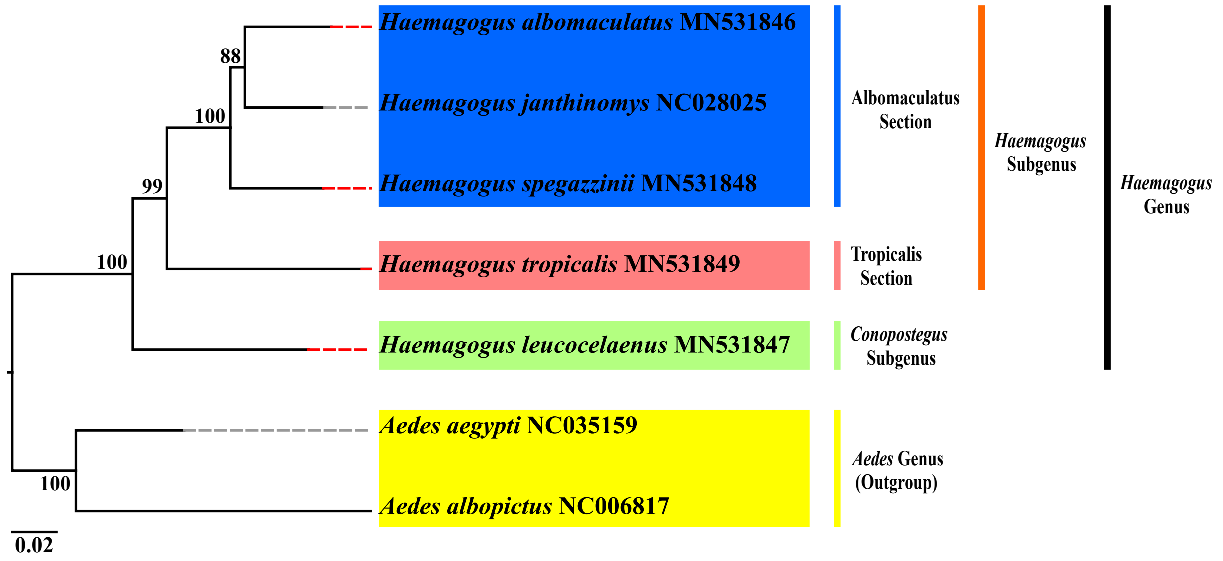

Supplementary Figure 7. Phylogenetic reconstruction by the Bayesian Inference method, based on the concatenation of the genes *ATP8*, *COI*, *ND5*, and *ND6* of the *Haemagogus* species sequenced in this study and 03 other taxa with data available on GenBank database. The subsequent Bayesian probabilities (BP) are shown on the left, in each node. The complementary lines dotted in red indicate the newly-sequenced species.

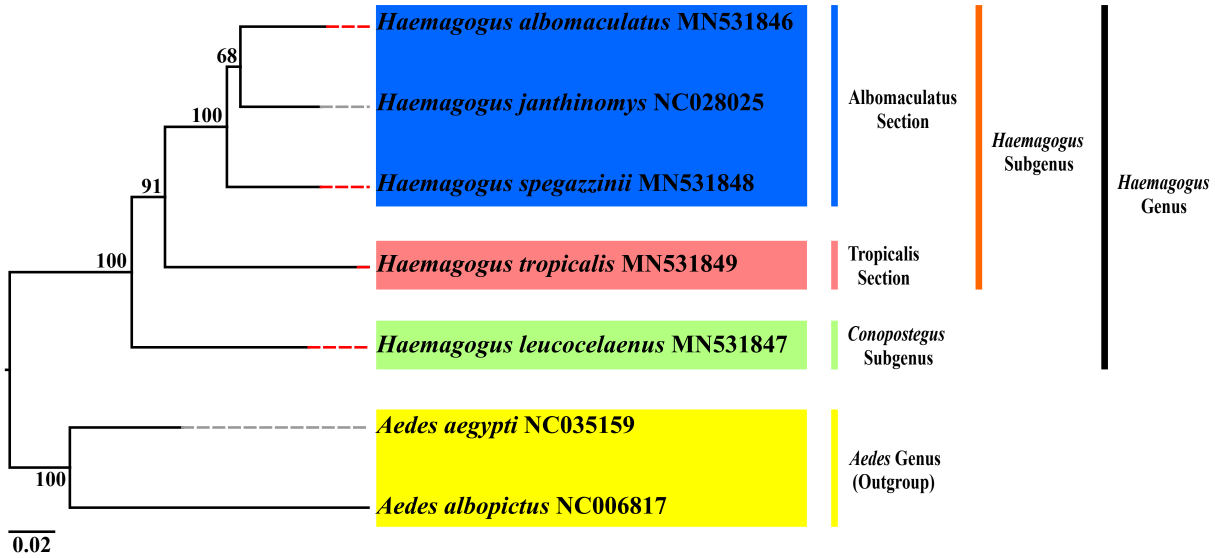

Supplementary Figure 8. Phylogenetic reconstruction by the Maximum Likelihood methods, based on the concatenation of the genes *ATP8*, *COI*, *ND5*, and *ND6* of the *Haemagogus* species sequenced in this study and 03 other taxa with data available on GenBank database. The values of support for bootstrapping (BPP) for 1,000 replicates are shown on the left, in each node. The complementary lines dotted in red indicate the newly-sequenced species.
